# The sympathetic nervous system in the 21st Century: neuro-immune interactions in metabolic homeostasis and obesity

**DOI:** 10.1016/j.neuron.2022.10.017

**Published:** 2022-11-02

**Authors:** Noelia Martinez-Sanchez, Owen Sweeney, Davi Sidarta-Oliveira, Alexandre Caron, Sarah A. Stanley, Ana I Domingos

**Affiliations:** 1 Department of Physiology, Anatomy and Genetics, University of Oxford, Oxford OX1 3PT, United Kingdom; 2 Physician-Scientist Graduate Program, Obesity and Comorbidities Research Center, School of Medical Sciences, University of Campinas, Campinas, Brazil; 3 Faculty of Pharmacy, Université Laval, Québec City, QC G1V 0A6, Canada; 4 Diabetes, Obesity and Metabolism Institute, Icahn School of Medicine at Mount Sinai, New York, New York 10029; 5 Department of Neuroscience, Icahn School of Medicine at Mount Sinai, New York, New York 10029

**Keywords:** sympathetic neurons, metabolism, adipose tissue, liver, pancreas, neuroimmunology, leptin

## Abstract

The sympathetic nervous system maintains metabolic homeostasis by orchestrating the activity of organs such as the pancreas, liver and white and brown adipose tissues. From the first renderings by Thomas Willis to contemporary techniques for visualization, tracing, and functional probing of axonal arborizations within organs, our understanding of the sympathetic nervous system has started to grow beyond classical models. In the present review, we outline the evolution of these findings and provide updated neuroanatomical maps of sympathetic innervation. We offer an autonomic framework for the neuroendocrine loop of leptin action, and we discuss the role of immune cells in regulating sympathetic terminals and metabolism. We highlight potential anti-obesity therapeutic approaches that emerge from the modern appreciation of SNS as a neural network vis the historical fear of sympathomimetic pharmacology, while shifting focus from post- to pre-synaptic targeting. Finally, we critically appraise the field and where it needs to go.

## Introduction

The Sympathetic Nervous System (SNS) is a branch of the Autonomic Nervous System (ANS) and is essential in regulating visceral function and various physiological processes. Anatomical dissections in the 17^th^ Century (first neuroanatomical map of sympathetic neurons was done by Thomas Willis, *Facsimili of Tabula X in Cerebri Anatome*, 1664, Bodleian Library, University of Oxford) (Willis, 1664) and subsequent ganglionic lesion studies gave a gross appreciation of SNS structure and function (du Petit, 1727, Stelling, 1867). Jacques Bénigne Winslow noted that these processes were activated ‘in response to distress or harm’, and thus termed them ‘Sympathetic’ (Winslow, 1740). Neurons innervating the same end organs and controlling antagonistic physiological processes were subsequently discovered and designated ‘Para-sympathetic’ (Langley, 1921). By the early-mid 20^th^ Century, a classical physiological dogma of the SNS initiating an acute physiological stress reaction (the so-called ‘fight or flight’ response) (Cannon, 1915) and the Parasympathetic Nervous System (PNS) working antagonistically to encourage processes of digestion and reproduction (so-called ‘rest and digest’) was established. However, experimental advances reveal nuances in SNS structure that question this dogma. Anatomical dissection is sufficient to identify large nerve bundles but falls short when describing smaller tracts and is poor at discerning the molecular identity of mixed nerves’ constituent neurons. Similarly, histology of sectioned tissues is poor in analysing tissues with a diffuse innervation pattern. Molecularly defining cell populations, accurate retrograde tracing and high-resolution 3D imaging techniques have all contributed to increasing the understanding of SNS anatomy and urged updating neuroanatomical maps. Likewise, the advent of cell-specific gene knockouts, viral retrograde tracing, chemogenetic and optogenetic control of specific cell populations and next-generation sequencing has better detailed the complexity underpinning autonomic regulation of peripheral tissues and challenged classical notions of autonomic function.

The canonical model of bivalent and oppositional autonomic function is inadequate in describing metabolic control, as some metabolic tissues receive innervation exclusively from sympathetic neurons, including brown adipose tissue (BAT) and white adipose tissue (WAT), and in others like the liver, the sympathetic innervation seems to play the crucial role in hepatic homeostasis. While the SNS has long been acknowledged as an essential arm of metabolic control, the modern experimental toolkit has afforded a more consolidated appreciation of the sympathetic contribution to metabolic homeostasis. In this review, we first outline how sympathetic neurons control the metabolic activity of the pancreas, liver, and adipose tissues. Then, we describe how these processes are related to immune function and dysregulated by the inflammatory responses that characterize metabolic diseases. Finally, we highlight emerging therapeutics targeting key sympathetic processes to reverse metabolic disease.

## Neuroanatomy of pancreatic sympathetic innervation

Sympathetic innervation is crucial for the regulation of pancreatic insulin and glucagon release and the control of glucose homeostasis, particularly by protecting against hypoglycemia during fasting and by increasing blood glucose during periods of elevated demand, such as by physical, inflammatory or psychological stress.

**Figure 1:**
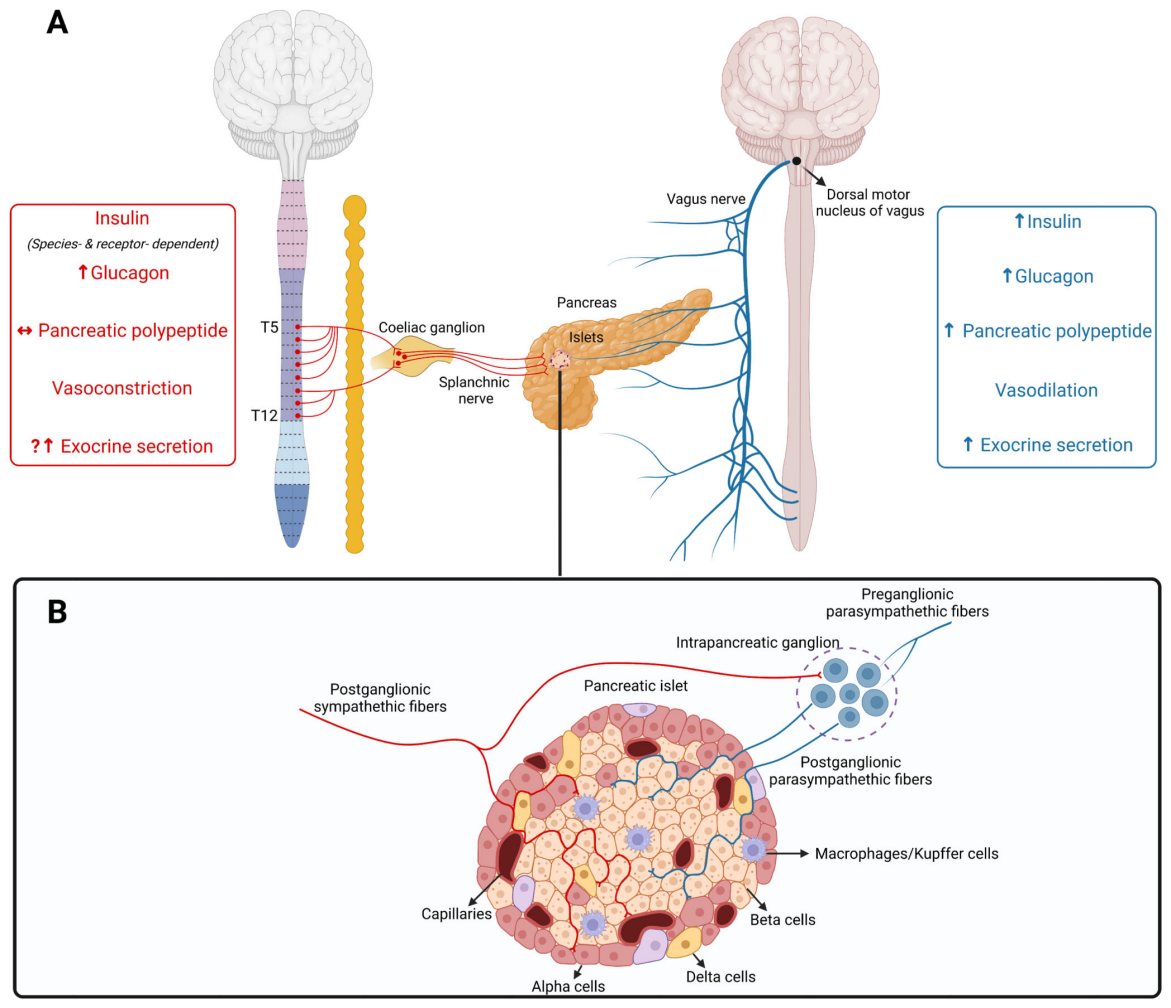
Efferent pancreatic innervation. **A**) The pancreas receives efferent sympathetic and parasympathetic innervation. Preganglionic sympathetic inputs arise from spinal levels T5-T12 projecting to the coeliac ganglia. Preganglionic parasympathetic inputs from the dorsal motor nucleus of the vagus project to the pancreas via the vagus nerve. The main reported results of sympathetic and parasympathetic stimuli are summarized in the left and right boxes. **B)** Postganglionic sympathetic fibres with en-passant boutons and terminal fibres to innervate pancreatic vasculature, intrapancreatic ganglia, exocrine and endocrine cells. Preganglionic parasympathetic fibres innervate a network of intrapancreatic ganglia. Postganglionic parasympathetic fibres innervate pancreatic vasculature, exocrine and endocrine cells.

Gross anatomical dissections and histological analyses of fixed pancreatic tissue, primarily in dogs and cats, provided an early anatomical framework for sympathetic, parasympathetic and somatic pancreatic innervation (Langerhans, 1937, Cajal and Sala, 1891) (Figure 1, **panel A**). Preganglionic sympathetic fibres in the greater thoracic splanchnic nerves provide inputs to coeliac ganglia neurons, which in turn provide postganglionic fibres to the pancreas. In addition, the vagus nerve provides preganglionic parasympathetic supply to a network of intrapancreatic ganglia. Neurons from the intrapancreatic ganglia then provide postganglionic cholinergic innervation to the exocrine and endocrine pancreas. Both vagal and spinal sensory fibres carry afferent sensory information from pancreas with their cell bodies in the nodose and dorsal root ganglia, respectively.

Methodological limitations in classic histology – namely poor stain tissue specificity and inadequate image resolution – led to conflicting reports regarding the distribution of sympathetic nerve fibres within pancreas (Cannon and Nedergaard, 2004). Improvements in neuron tracing, staining, and imaging have provided greater clarity about the anatomy of sympathetic pancreatic innervation. Sympathetic innervation of the pancreas was visualised by early fluorescence microscopy studies in the pancreata of rats, mice, chickens, guinea pigs, baboons, zebrafish (Holst et al., 1981, Kamel et al., 1979, Liu et al., 1997, Ulas et al., 2003, Verchere et al., 1996, Yang et al., 2018) and humans (Jarhult and Holst, 1979). Dense sympathetic projections to pancreatic islets (Hisatomi et al., 1985, Rodriguez-Diaz et al., 2011) as well as acinar tissue, ducts (Ulas et al., 2003) and blood vessels (Lindsay et al., 2006) were identified, defining four types of pancreatic plexuses: peri-insular, periacinar, periductal and perivascular. Higher resolution electron microscopy (EM) imaging confirmed the existence of such plexuses and revealed that sympathetic innervation is largely comprised of fine unmyelinated fibres that are lost after chemical sympathectomy 6-hydroxydopamine (6-OHDA) (Ahren et al., 1981, Sunami et al., 2001, Ushiki and Watanabe, 1997, Chien et al., 2016).

Despite high resolution imaging, studies that required tissue sectioning may not provide a representative sample of neurons across a diffusely innervated organ such as the pancreas. Studies utilising confocal microscopy of fluorescently-labelled tyrosine hydroxylase (TH)+ sympathetic fibres allowed for 3D reconstruction and quantification of sympathetic innervation across the entire pancreas. In mice, such studies showed TH+ fibres distributed evenly throughout the pancreas (Lindsay et al., 2006) and the density of sympathetic innervation is equal in the endocrine and exocrine pancreas. Furthermore, a significantly greater number of contacts between sympathetic fibres and glucagon-secreting alpha cells than insulin-secreting beta cells (Rodriguez-Diaz et al., 2011), reflecting catabolic and anabolic functions of these hormones respectively, were also reported (Figure 1, **panel B**). Still, tissue sectioning an entire organ is laborious and tracing filamentous structures across serial sections is both time consuming and prone to artefacts. Tissue clearing, light sheet microscopy and image analysis have enabled the best 3-dimensional imaging and quantification of pancreatic innervation to date. Studies applying these methods in mice highlight sympathetic innervation to intrapancreatic ganglia, islets and vasculature (Tang et al., 2018b). Sympathetic fibres closely follow vasculature, forming both peri-vascular plexuses that innervate capillaries in the islet core, and peri-insular plexuses that contacts alpha cells (Tang et al., 2018b, Chien et al., 2019, Chiu et al., 2012, Tang et al., 2014) (Figure 1, **panel B**). Quantification of TH+ innervation across hemipancreata confirmed relatively even distribution through the pancreas but suggested TH+ innervation is enriched in mouse islets compared to the exocrine pancreas, representing 3% of islet volume (Alvarsson et al., 2020).

While most studies demonstrate dense sympathetic innervation in human pancreatic tissue, there are reported species differences between humans and rodents. However, there are several confounding factors that should be considered for almost all histological studies examining human pancreata and that account for differences between the mentioned observations. Human tissue samples are difficult to attain, especially for pancreatic tissue, where the presence of strong autolytic digestive enzymes means biopsies are not routinely performed. Rare surgical specimens of human pancreatic tissue are usually from individuals with significant pancreatic disease such as chronic pancreatitis or pancreatic cancers. As such, most human pancreatic tissue is obtained post-mortem and differences in patient comorbidities, treatment regimes, tissue processing and the presence of extensive digestive enzymes in pancreatic tissue may lead to significant variability between samples. Despite these limitations, detailed confocal imaging and quantification in cryosections from human pancreatic tissue identified sympathetic fibres in exocrine pancreas and islets (Rodriguez-Diaz et al., 2011) . In human islets, sympathetic fibres contacted vascular smooth muscle cells. The few contacts on endocrine cells were reported with somatostatin-releasing delta cells. Later studies using tissue-clearing and 3D imaging in human pancreatic tissue suggest sympathetic innervation of alpha, beta and delta cells (Chien et al., 2019, Tang et al., 2018a, Campbell-Thompson et al., 2021) as well as TH^+^ fibres adjacent to intralobular adipocytes (Chien et al., 2019). Given the small sample size of many human pancreatic imaging studies, attempts to reproduce existing data would be useful to minimise effects of inter-sample variability.

Initial gross dissection studies that sought to map the central and peripheral sympathetic inputs to pancreas identified the coeliac and mesenteric ganglia as providing postganglionic sympathetic innervation to the pancreas. This observation was bolstered by absence of TH+ cell bodies in intrapancreatic ganglia (Oomori et al., 1994), by significant reductions (90%) in pancreatic catecholaminergic innervation after coeliac and mesenteric ganglionectomy (Anglade et al., 1987), and by pancreatic non-viral retrograde tracing studies that visualized inputs from coeliac, splanchnic and mesenteric ganglia (Quinson et al., 2001). Pseudorabies virus (PRV) retrograde tracing studies allowed mapping the central circuits controlling pancreatic sympathetic inputs, identifying pancreas-projecting neurons in the spinal cord, brain stem and multiple hypothalamic regions (including the arcuate (ARC), paraventricular (PVH), ventromedial (VMH) and dorsomedial (DMH) nuclei) (Buijs et al., 2001, Rosario et al., 2016, Jansen et al., 1997). These findings are in keeping with earlier studies that found hypothalamic lesions in the VMH, DMH and PVH decreased splanchnic nerve activity (Yoshimatsu et al., 1984). More recent studies using pharmacological, genetic and neuromodulatory approaches have begun to dissect the contribution of specific hypothalamic nuclei to the control of pancreatic sympathetic innervation.

Chan et al demonstrated that VMH infusion of muscimol, a GABA receptor agonist, during a hypoglycemic clamp in rats significantly blunted the release of pancreatic glucagon (Chan et al., 2006). In keeping with a role for VMH neurons, knockout of glutamate transporter, VGLUT2, in steroidogenic factor-1 (SF1) neurons (a major subset of VMH neurons) impairs the pancreatic glucagon response to fasting leading to hypoglycemia (Tong et al., 2007). These studies are supported by further work using neuromodulation of VMH neurons. Optogenetic silencing of VMH SF1 neurons (Meek et al., 2016), or VMH neurons expressing neuronal nitric oxide synthase (Faber et al., 2018) significantly blunted the glucagon response to hypoglycemia. In addition, recent work suggests PVH oxytocin neurons suppress pancreatic insulin secretion via modulating sympathetic activity (Papazoglou et al., 2022). Together these studies provide evidence of a polysynaptic pathway from hypothalamic nuclei via the brain stem and abdominal ganglia to regulate pancreatic sympathetic activity. Further work investigating central nervous system (CNS) control of the endocrine pancreas has been recently reviewed in Faber *et al* (Faber et al., 2020). Although there has been progress in determining the precise roles of some hypothalamic circuits in regulating pancreatic sympathetic output, the contributions of additional hypothalamic and extra-hypothalamic regions and the specific neural populations involved in these circuits remain to be determined. Similarly, studies examining functional and structural alterations of these central pathways in disease are scarce.

Studies assessing the physiological roles of pancreatic sympathetic innervation have used electrical nerve stimulation, denervation, and pharmacology, to identify significant effects on blood flow, pancreatic hormone release and pancreatic anatomy secondary to sympathetic signalling. Activation of pancreatic sympathetic innervation releases noradrenaline (NA), together with neuropeptides such as galanin (Scheurink et al., 1992) and neuropeptide Y (NPY) (Carlei et al., 1985, Su et al., 1987). Pancreatic islets express α-2, β-2 and β-3 adrenergic receptors. Vascular tone, hormone release and exocrine function are the main factors controlled by sympathetic supply in the pancreas. Pancreatic vascular tone has been shown to be partly controlled by sympathetic supply. Electrical stimulation of the sympathetic preganglionic splanchnic nerve or postganglionic pancreatic nerve leads to rapid (within 30s) vasoconstriction in the pancreata of cats, dogs, mice and rats (Brooksby and Donald, 1971, Kurose et al., 1990, Porte et al., 1973, Richins, 1953, Almaca et al., 2018). In mouse models and living pancreas slices from human donors, sympathetic agonists stimulate contractile pericytes leading to vasoconstriction (Almaca et al., 2018, Mateus Goncalves and Almaca, 2020). Changes in islet blood flow (IBF) provide an additional mechanism for sympathetic regulation of glucose homeostasis beyond direct signalling to the islets (Atef et al., 1992, Jansson and Hellerstrom, 1986). Hyperglycemia results in islet capillary dilation and redistribution of IBF leading to increased insulin release through central parasympathetic signalling (Jansson and Hellerstrom, 1986). Conversely, sympathetic signalling via α-2 receptor agonists leaded to vasoconstriction, decreased IBF and reduced insulin release (Atef et al., 1992, Jansson and Hellerstrom, 1986). However, conflicting evidence exists regarding the different adrenergic receptors regulating IBF and insulin secretion. Most studies stimulate a single receptor as a model of native sympathetic signalling when in reality several receptors are concomitantly and differently targeted by sympathetic neurons and circulating adrenaline levels. While α-receptor signalling has been shown to decrease IBF and insulin secretion (Almaca et al., 2018, Atef et al., 1992), the opposite has been demonstrated for β-receptors, in particular for β-3 receptor signalling, which increases IBF and insulin secretion in rats (Atef et al., 1996). The precise function and the relative activation of either set of receptors by direct sympathetic synapses and by circulating noradrenaline and adrenaline levels are yet to be determined. An additional confounder when studying the paired control of vascular and endocrine function in the pancreas is the use of different animal models of disease. For instance, α-2 and β-3 signalling has been shown to have similar effects in Goto-Kakizaki rats - a nonobese type 2 diabetes model with higher IBF in comparison to control rats. In this model, treatment with either α-2 antagonists (Sandberg et al., 2013) or with β-3 antagonists (Pettersson et al., 2009) reduced IBF. The effects of sympathetic stimulation on insulin release are somewhat contradictory. Several studies report splanchnic nerve stimulation decreased basal and glucose-stimulated insulin release in dogs and rats (Kurose et al., 1990, Ahren et al., 1987, Girardier et al., 1976, Roy et al., 1984). However, there are also reports that splanchnic nerve stimulation increased in insulin release in dogs and pigs, possibly secondary to hyperglycemia (from increased glucagon) with splanchnic stimulation (Holst et al., 1981, Kaneto et al., 1975). Differences are also seen with sympathetic denervation that decreased plasma insulin in mice but increased plasma insulin in rats (Sunami et al., 2001). Pharmacological studies also show disparities. NA infusion into the pancreas had no effect on basal insulin release at any dose in dogs (Ahren et al., 1987). In contrast, a study by Kaneto et al suggested α- and β-adrenergic receptors do influence insulin release as beta blockade reduced basal insulin release while alpha antagonists increased insulin (Kaneto et al., 1975). In rats, α-2 adrenergic receptor antagonist treatment reduced glucose-evoked insulin release while α-1 and β-adrenergic receptor antagonists had no effect (Niddam et al., 1990).

Studies examining the roles of pancreatic sympathetic nerves on glucagon release have been more consistent. Splanchnic and pancreatic nerve stimulation increased pancreatic glucagon release into either the portal or systemic circulation in many species, including humans (Jarhult and Holst, 1979, Esterhuizen and Howell, 1970, Marliss et al., 1973). In some species, the increase in glucagon release with nerve stimulation is glucose dependent, with greater increments in glucagon secretion with nerve stimulation at low glucose concentration (Holst et al., 1981, Kurose et al., 1990). This is in line with the reported sympathetic response to insulin-induced hypoglycemia by increasing glucagon secretion (Mundinger et al., 2016). In keeping with the response to nerve stimulation, chemical sympathetic denervation with 6-OHDA reduced basal and stimulated glucagon release in rats (Hisatomi et al., 1985, Lindquist and Rehnmark, 1998). Pharmacological studies mostly reproduce the effects of sympathetic nerve stimulation on blood flow and glucagon release. NA infusion into dogs reduced IBF and increased glucagon release, but this effect was dose-dependent and NA infusion at the highest concentration had the opposite effect (Ahren et al., 1981), which can arguably be attributed to differential signalling through alpha- and beta-receptors. Pharmacological studies also suggest that both alpha- and beta-adrenergic receptors regulate pancreatic glucagon release. Beta blockade with propranolol reduces basal glucagon release but has no effect on the glucagon response to electrical nerve stimulation, while alpha adrenergic blockade had no effect neither on basal glucagon output nor in its release upon electrical nerve stimulation (Kaneto et al., 1975). Other ex-vivo studies found contradictory results, in which glucagon release upon electrical nerve stimulation was completely abolished by alpha-adrenergic blocade (Kurose et al., 1990). However, there is also evidence that non-adrenergic neurotransmission may contribute to sympathetic regulation of glucagon release. In one study, combined α- and β-adrenergic receptor blockers did not block the increase in glucagon release with sympathetic nerve stimulation suggesting neuropeptide release from sympathetic nerves may also contribute to glucagon release (Dunning et al., 1988).

However, splanchnic nerve stimulation and pharmacological studies do not specifically target pancreatic innervation, and it is possible that effects on other organs innervated by the coeliac ganglia, such as the liver, or modified by systemic adrenergic receptor antagonists may indirectly alter pancreatic insulin release. An additional challenge is discerning which changes to beta cell function are due to direct sympathetic neuronal signalling, and which are secondary to changes in islet blood flow, as evidence amounts for the parallelism of insulin secretion and vasodilation (Richins, 1953, Almaca et al., 2018, Atef et al., 1992, Jansson and Hellerstrom, 1986, Atef et al., 1996, Pettersson et al., 2009). Furthermore, whether the observed effects upon pharmacological blockade are related to neuronal signalling remains unknown - contrary to the CNS, the autonomic nervous system has been traditionally studied by targeting its downstream receptors rather than selective activation of specific circuits. Further studies with highly targeted modulation of pancreatic innervation are needed to dissect the contribution of sympathetic signalling to pancreatic hormone release. In particular, beyond optogenetic strategies, studies performing selective genetic ablation of specific adrenergic receptors in both pericytes and beta cells are needed to precisely determine the direct contribution of sympathetic signalling to insulin and glucagon release, and to further separate these effects from those related to circulating adrenaline and NA.

Some studies have assessed whether sympathetic innervation also regulates the exocrine functions of the pancreas. Early studies in guinea pigs suggested so since NA administration potentiated cholecystokinin-induced amylase release (Joehl et al., 1983). However, further studies found that sympathectomy has little effect on the innervation of acinar cells, indicating that sympathetic effects on exocrine function are indirect through vasoconstriction and reduction of blood flow. Yet, a recent study found that blocking beta signalling with systemic propranolol administration led to reduced absorption of dietary fat by inhibiting the expression of lipase, for which a mechanism involving cyclic AMP response element binding protein (CREB) was described (Baek et al., 2018), thus providing an additional axis by which sympathetic signalling regulates pancreatic function and response to obesogenic stimuli. Very few studies followed, and thus not much is known about possible pathways that project from the CNS to the pancreas via sympathetic signalling. Instead, most studies have examined the effects of pancreatic sympathetic innervation on its endocrine function, probably due to its much greater medical significance.

Recent intriguing work has suggested that pancreatic sympathetic innervation may play a role in the development, maintenance and remodelling of islet structure. Genetic or pharmacological ablation of sympathetic innervation in mice altered pancreatic islet structure reduced plasma insulin and impaired glucose tolerance. The effects on islet structure were partly reversed by beta-adrenergic receptor agonists (Borden et al., 2013). In addition, both alpha-1 and alpha 2 adrenergic receptor antagonists were able to blunt beta cell proliferation induced by nutrient infusion (Moulle et al., 2019). These findings suggest sympathetic signalling may have effects on islet structure as well as regulating the function of pancreatic endocrine cells.

Unlike the liver or adipose tissue, the pancreas receives dense parasympathetic innervation and islet expression of cholinergic receptors M2 and M3. Studies using electrical stimulation of the efferent vagus nerve lowered blood glucose (Meyers et al., 2016) while activating parasympathetic inputs to transplanted islets increased insulin secretion (Rodriguez-Diaz et al., 2012). The effects of vagal stimulation on insulin release were blocked by hexamethonium suggesting nicotinic receptor mediated effects at intrapancreatic ganglia (Ahren and Taborsky, 1986). Parasympathetic postganglionic cholinergic fibres innervate beta cells and stimulate insulin release, largely through muscarinic receptors (Karlsson, 1993). Therefore, the major actions of pancreatic parasympathetic nerves appear to oppose the effects of pancreatic sympathetic innervation, in line with the classic antagonistic doctrine of autonomic nervous system. However, nerve stimulation and pharmacological studies lack organ specificity, and further work with organ specific neuromodulation may reveal new roles for pancreatic sympathetic and parasympathetic innervation.

In keeping with findings in other organs, there is evidence of altered sympathetic innervation in the pancreas with altered nutrition, diabetes, leptin deficiency and impaired leptin signalling and with the obese phenotype in general. Malnutrition increased sympathetic activity in the superior cervical ganglion in rats and reduced glucose-stimulated insulin release (Leon-Quinto et al., 1998). Conversely, acute hyperglycaemia induced by glucose infusion reduced pancreatic sympathetic activity and increased basal and glucose- stimulated insulin release in vivo (N’Guyen et al., 1994). The effects of long-term over-nutrition are less clear. In obese Zucker rats with leptin receptor defects both pancreatic NA content and turnover are reduced (Levin et al., 1981), and insulin hypersecretion was related to impaired sympathetic signalling (Lee et al., 1993). In wild-type Sprague-Dawley rats prone to diet-induced obesity (DIO), lower pancreatic NA turnover was associated with hyperinsulinemia and increased body fat (Levin et al., 1987, Levin et al., 1983) in comparison to their obesity-resistant counterparts. In agreement with these observations and with the central control of these stimuli at the hypothalamus, NA content and turnover were also reduced in the VMH in rats prone to DIO when compared to those resistant to it. However, in *db/db* mice, recent work reported remodelling of pancreatic ducts close to islets and increased sympathetic innervation of the islet-duct boundary (Tang et al., 2018b). Increased sympathetic innervation in the neuro-insular plexus was also reported in *db/db, ob/ob* mice and in wild-type mice fed a high fat diet - remarkably, in *db/db* mice, this observation was correlated with increased age and progression of the diabetic phenotype (Giannulis et al., 2014). However, the changes in the sympathetic innervation in mouse models of obesity as metabolic disease progresses have not been systematically assessed, so it remains unclear if the increased sympathetic innervation and reduced activity occur together. It is possible that sympathetic fibre density may increase to compensate for reduced pancreatic sympathetic activity, but further studies are needed to understand these contradictory findings.

Even less evidence is available regarding human obesity, and the remarkably reduced autonomic innervation to islets in the human pancreas in comparison to rodents ought to lead to prominent changes in how changes in sympathetic input impact the development of obesity and its comorbidities.

## Autonomic innervation of the liver

In 1849, Claude Bernard, noted pricking the floor of the fourth ventricle in rabbits was sufficient to increase hepatic glucose production and that transection of the splanchnic (sympathetic) nerves, but not the vagus branch innervatin the liver (parasympathetic), would inhibit this effect (Bernard, 1854). Following Bernard’s functional studies, innervation of the liver started to be imaged in the latter half of the 19^th^ century using mainly osmium, silver and gold staining techniques. Early studies described a profuse innervation primarily associated with the vessels and ramifications amongst parenchymal cells (Riegele, 1928). Electron microscopy studies captured images of sufficient resolution to perform better structural analysis on hepatic innervation, showing that neurons would terminate on hepatocytes adjacent to connective tissue of the portal triads in the mouse liver (Yamada, 1965). In addition, cytostructural analysis showed that some terminals contain vesicles with a dense core – a hallmark of catecholamine-containing vesicles (Yamada, 1965).

More reliable analysis of cellular identity came with the advent of histochemical methods, but early studies suffered from poor specificity and an appreciable degree of intra-model variability. In particular, the relatively diffuse 3D innervation pattern was poorly captured by histological slicing methods. Early studies utilising acetylcholinesterase (AchE) specific staining suggested cholinergic hepatic input to hepatic vessels, bile ducts and intralobularly in close apposition to hepatocytes and sinusoids (Skaaring and Bierring, 1976, Sutherland, 1964). In models of *T. Belengeri*, Forssman and Ito used fluorescence microscopy to show sympathetic nerve fibres in close apposition to hepatocytes in the peripheral zone of the lobule where parenchymal cells, sinusoidal lining cells and Kupffer cells also appeared to be innervated (Forssmann and Ito, 1977). Auto-radiography measurements of exogenously administered [^3^H]-noradrenaline to hepatic nerves suggested that they were predominantly sympathetic, and chemical sympathectomy with 6-OHDA caused degeneration of intralobular nerve fibres. While less abundant, similar liver innervation was reported in *Macaca Mulatta* (Forssmann and Ito, 1977). However, these data were disputed by Reilly and colleagues who failed to find an intralobular plexus of nerves in rat liver when adrenergic and acetylcholinaminergic histochemical stains were applied - instead, nerves were found to be contiguous to branches of the portal vein and hepatic artery, suggesting that the effects of hepatic adrenergic signalling are solely mediated by the regulation of vascular tone (Reilly et al., 1978).

**Figure 2:**
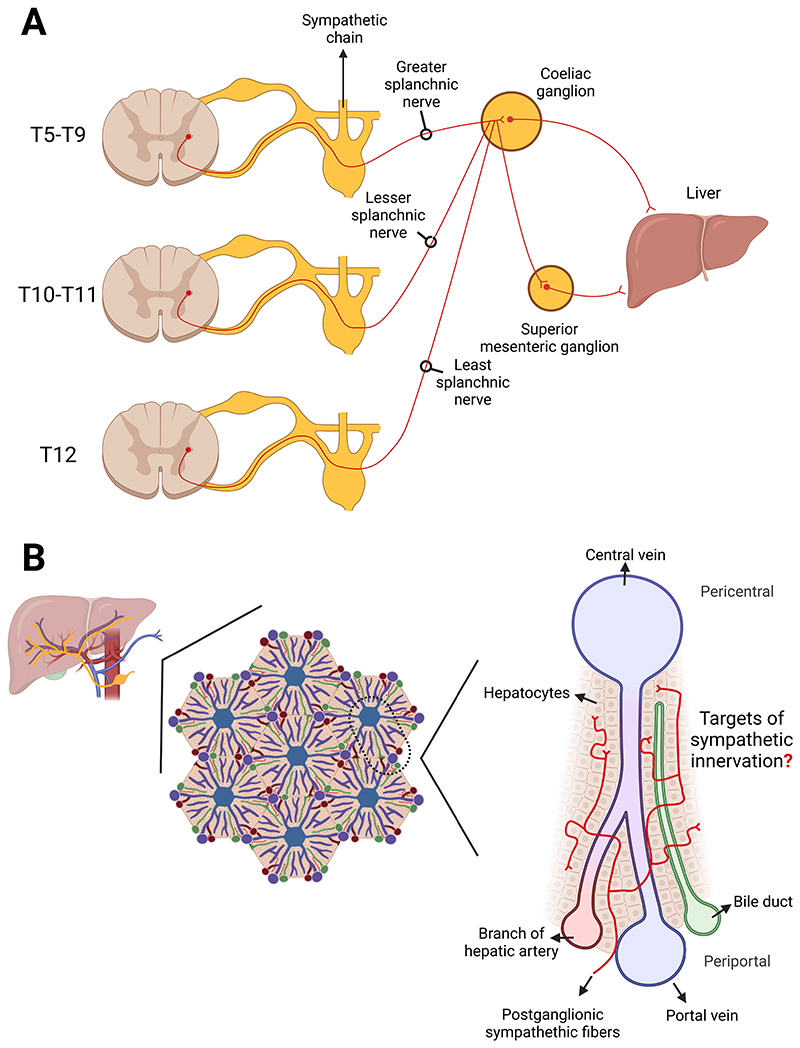
Sympathetic Innervation of Liver. **A)** Postganglionic neurons projecting to the main lobe of the liver were identified in medial ganglia including the celiac-superior mesenteric complex. **B)** Sympathetic nerve fibres enter the liver along the portal vein. Nerve plexuses were identified in the hepatic triads, particularly around the branches of the hepatic arteries. From these nerve plexuses, branches enter the hepatic lobule in the space of Disse and extend towards the central vein to outline the hepatic sinusoids. These nerve fibres run primarily around the portal vein, hepatic artery and bile duct and it is debated whether they make direct contact with hepatocytes in the peripheral zone of the lobule, parenchymal cells, sinusoidal cells, and Kupffer cells.

Retroviral PRV tracing studies have mapped the neuroanatomical origin of hepatic sympathetic nerves. Two studies report sympathetic neurons originating from the coeliac superior mesenteric complex, aorticorenal and suprarenal ganglia, which are in turn innervated by splanchnic preganglionic neurons located in the intermediolateral column of the spinal cord (T7–T12) (Yi et al., 2010, Torres et al., 2021) (Figure 2). Perhaps some caution should be taken in interpreting these results – both studies injected PRV only in the median lobe of the liver, which may not be entirely representative of the whole-liver innervation pattern.

### Functional studies on the neural control of liver metabolism

Since Claude Bernard’s early lesion studies, a more consolidated appreciation on the function of hepatic autonomic innervation has been sought. In the 1960s, Shimazu and colleagues established that electrical stimulation of rabbit splanchnic nerve was sufficient to increase the hepatic gluconeogenetic enzymes glycogen phosphorylase and glucose-6-phosphatase (Shimazu and Fukuda, 1965). Furthermore, they found the stimulatory effect on glycogen phosphorylase was still present after adrenalectomy or pancreatectomy, indicating neither adrenal hormones nor pancreatic glucagon mediated these effects. Electrical stimulation of the splanchnic nerves in perfused liver models also induces physiological changes that align with acute stress response – altered blood flow, increased glycogenolysis and glycolysis, decreased urea cycle activity (Beuers et al., 1986, Niijima and Fukuda, 1973, Gardemann et al., 1992), decreased ketogenesis (Gardemann et al., 1992) and increased plasma glucose in humans and rodents. These hepatic hyperglycaemic effects are mimicked by selective alpha-1 adrenoceptor agonism in isolated rat liver parenchymal cells (Hutson et al., 1976). Similarly, systemic noradrenaline injection rapidly mobilizes glucose from the liver by increasing hepatic glucose production (Puschel, 2004). Overall, autonomic regulation is a crucial aspect of normal hepatic function, being involved in glucose and lipid metabolism and implicated in metabolic disease - nevertheless, similarly to studies exploring the pancreas, most approaches are based on the systemic administration of pharmacological agonists or inhibitors to modulate downstream receptors, and the precise circuitry involved in its signalling from the CNS and in the periphery are mostly unknown. New approaches combining retrograde tracing, three-dimensional mesoscopy and chemo- and opto-genetics manipulation of hepatic SNS supply ought to shine light on the neuronal control of liver function in health and disease.

Despite the evidence that the liver is strictly innervated by the sympathetic nervous system, recent functional studies defend different perspectives. In one study, it was shown that surgical resection of the hepatic branch of the vagus nerve in rats would prevent the effects of brain-infused insulin on hepatic glucose production (Pocai et al., 2005). Since this nerve bundle comprises both afferent and efferent nerves, it is possible that sensory vagal signals, which were suggested to sense glucose levels, were affected by the mechanical denervation (Kwon et al., 2020). Consistently, the authors also performed vagal deafferentation by resecting the vagal branch at the site of entry to the brainstem and observed that this procedure would not modify how brain-infused insulin would suppress glucose production - further supporting that distal sensory nodose neurons would mediate the effect. Although it remains possible that the vagal deafferentation procedure was not complete, the authors defend that the rat liver contains parasympathetic innervations. Immunolabeling studies of whole rat liver are therefore warranted to confirm whether this species is different from mice and non-human/human primates in terms of liver innervation.

Another study used optogenetic approaches to identify the circuit by which pro-opiomelanocortin (POMC) neurons control blood glucose (Kwon et al., 2020). In a first set of experiments, they injected viral particles expressing a wheat germ agglutinin (WGA) and Cre recombinase fusion protein into the liver of C57BL/6J mice. This system allows for anterograde transneuronal transport (Libbrecht et al., 2017). They also injected the same animals with AAV expressing Cre-dependent ChR2 linked to a neuronal POMC enhancer in the ARC. They then performed optogenetic stimulation of the dorsal nucleus of vagus nerve (DMV) for one hour and reported increased blood glucose together with increased expression of gluconeogenic genes in the liver and a better ability for pyruvate to increase blood glucose. In a second set of experiments, they injected a retrograde vector expressing a Cre-dependent inhibitory opsin Jaws allowing in the liver of ChAT-IRES-Cre mice. They then mounted LED modules of the abdomen to inhibit the choline acetyltransferase (ChAT) fibers through light penetration across the skin and reported increased blood glucose. Since this was accompanied by increased expression of gluconeogenic genes in the liver, they concluded increased gluconeogenesis was responsible for the effects on blood glucose. Although the authors provided a thoughtful discussion on the inconsistencies with the prior findings (Kwon et al., 2020), it remains hard to reconcile the findings with the convincing anatomical studies showing no parasympathetic innervation in the mouse liver (Liu et al., 2021, Fujikawa et al., 2010). One explanation is that the approach may have affected *en passant fibres* related to the sympathetic nervous system, impacted afferent signals, or have influenced surrounding organs, such as the adrenal glands or the pancreas. Even though the authors show no changes in corticosterone and glucagon one hour after the illumination, blood glucose increased quickly (within 15 min) and it is possible that hormone levels were affected in the first minutes.

## Sympathetic innervation of adipose tissues

### Brown Adipose Tissue

**Figure 3:**
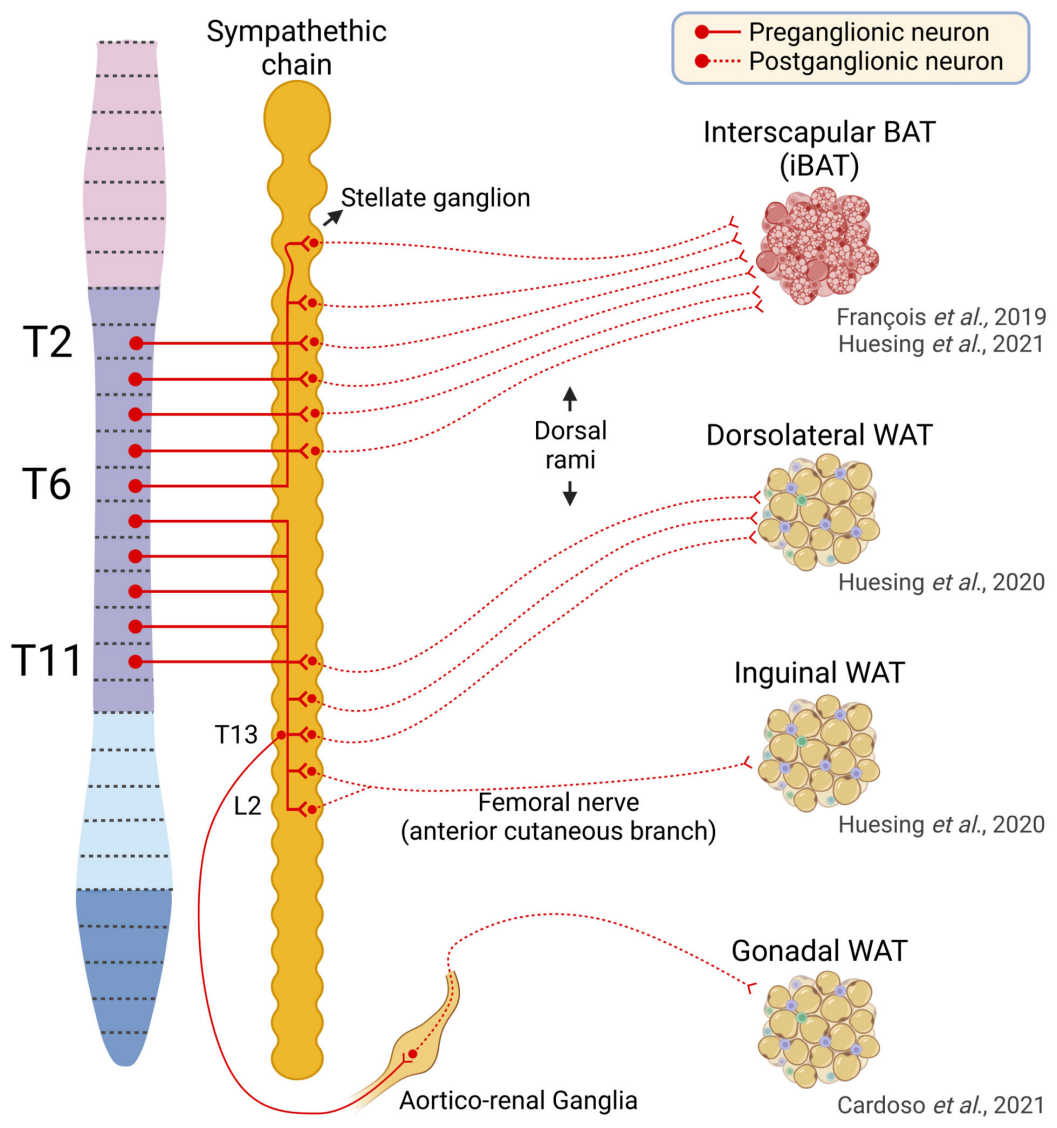
Neuroanatomy of sympathetic innervation of Adipose tissues. Retrograde poly-synaptic tracing of sympathetic circuity with pseudorabies virus (PRV) ^GFP^, visualised after clearing of whole murine mediastini of TH-CreR26dtTomato reporter mice showed that interscapular BAT (iBAT) receives preganglionic input originating from spinal levels T2-T6, and post-ganglionic fibres from the stellate ganglia and T1-5 paravertebral ganglia. The continuous white fat pad that extends from the dorsolateral to the ventromedial inguinal region (inguinal WAT) is anatomically divided into ‘dorsolateral’ WAT (dlWAT) and ‘inguinal’ WAT (iWAT) by the superficial ilium circumflex vein. Studies analysing this fat depot using a similar retrograde tracing approach show preganglionic innervation to the iWAT arising from spinal levels T7 to T11. dlWAT receives postganglionic innervation from T11-T13 paravertebral ganglia, whereas iWAT receives innervation possibly from the anterior cutaneous branch of the femoral nerve, originating from L1-2 sympathetic chain neurons that merge onto the lumbar plexus. Retrograde tracing with GFP-labelled adeno-associated virus shows that gonadal WAT (gWAT) receives sympathetic innervation from the aortic renal ganglion at the level of T13, but the spinal level from which these fibres originate is not known. (Solid line represents preganglionic innervation and dashed line represents postganglionic innervation).

Brown Adipose Tissue (BAT) performs non-shivering thermogenesis by dissipating the mitochondrial H+ gradient across the mitochondrial inner membrane while circumventing ATP synthase. Metabolic substrate is thus oxidised without generating chemical stores of energy, instead generating heat (Figure 3). BAT is well adapted for this function, expressing uncoupling protein 1 (UCP1), containing multilocular adipocytes, a high density of mitochondria for heat generation and a rich capillary network for heat dissipation.

BAT was recognised as relevant to the physiology of rodents and human infants as their large surface area to volume ratios required additional support in maintaining core body temperature (Cannon and Nedergaard, 2004). Only with advances in positron emission tomography (PET) combined with computed tomography (CT) (PET-CT) imaging and immunohistochemistry has its presence been shown in adult humans (Cypess et al., 2009, Lidell et al., 2013). While distributed somewhat heterogeneously, in adult humans BAT tends to predominate in subcutaneous neck, supraclavicular, axillary, and paravertebral regions (Ikeda et al., 2018).

BAT has an impressive ability to use energy – it consumes more glucose per gram than any other peripheral tissue when stimulated, except perhaps the brain (Orava et al., 2011) and estimates suggest that 50g of maximally stimulated brown adipose tissue could account for up to 20% of daily energy expenditure in an adult human (Rothwell and Stock, 1983). In addition, BAT appears to confer protection against metabolic syndromes - mouse strains with greater BAT have a correlated lower susceptibility to diabetes and obesity (Guerra et al., 1998), and ectopic BAT deposits in skeletal muscle protect mice against diet-induced obesity (Almind et al., 2007). Understanding the control mechanisms and nature of the changes underlying control of BAT thermogenesis is of much interest.

Sympathetic innervation has emerged as an important regulator of BAT thermogenesis - since the initial descriptions of the central role of sympathetic stimulation for maintenance of body temperature upon cold exposure (Maickel et al., 1967) and its interrelations with the hypothalamus-pituitary-adrenocortical axis (Fukuhara et al., 1996), sympathetic control of thermogenesis and energy expenditure has become of great interest, in particular due to its therapeutic potential against obesity. However, the poor specificity and sensitivity of early histological stains meant early fluorescence microscopy studies were conflicted on the existence (Wirsen, 1964, Wirsen, 1965) of BAT sympathetic innervation, and whether adipocytes, parenchyma or vessels received innervation. As techniques improved, it emerged that BAT received a substantial sympathetic supply (Foster et al., 1982a, Foster et al., 1982b). Recent iDISCO whole tissue clearing and PRV retrograde tracing studies show that interscapular BAT (iBAT), the largest brown depot in mice, receives sympathetic preganglionic innervation from T2-6 and post-ganglionic projections from caudal stellate ganglion (T1) and T2-5 sympathetic chain ganglia (Francois et al., 2019) (Figure 3). Using similar approaches in TH reporter mice has granted better resolution and clarified that iBAT receives innervation from dorsal rami (not intercostal nerves), possesses strong differential and preferential sympathetic innervation of BAT over WAT in the interscapular region, and forms neuron-adipocyte junctions from sympathetic varicosities (Huesing et al., 2022). The use of transgenic animals and central analysis of viral retrograde tracers is not possible in humans, and thus the neuroanatomy of human BAT tissue remains poorly described.

Sympathetic signalling is a strong driver of BAT thermogenesis. Surgical sympathectomy or pharmacological ganglionic blockade of BAT in rodent models decreases metabolic markers (lipoprotein lipase activity, thermogenic mitochondrial protein activity, glucose membrane transporter density), increases weight without affecting feeding behaviours and inhibits physiological responses to cold challenges. In denervated subjects - all of these processes can be acutely resolved via NA injection (Bartness and Wade, 1984, Benzi et al., 1988, Rothwell and Stock, 1984, Takahashi et al., 1992). More recently, optogenetic stimulation of sympathetic neurons innervating mouse iBAT was sufficient to induce thermogenesis, increasing core body temperature and *Ucp1* mRNA (Lyons et al., 2020), but these authors did not record or report any changes in body weight. If optogenetic stimulation of BAT were to reduce weight and improve metabolic markers, this could provide powerful evidence that BAT activation is sufficient to improve metabolic state.

Understanding structural, functional, and molecular changes associated with upregulated BAT thermogenesis could help unearth important pathways for potentiating its activity in humans. Imaging studies utilizing sectioning methods reported that cold exposure increases sympathetic nerve density and arborisation of BAT (Murano et al., 2009, De Matteis et al., 2013) but modern quantitative analysis of the whole organ would offer better descriptions of tissue innervation following a cold challenge. Interestingly, Blaszkiewicz et al used this technique to study adipose innervation in response to cold exposure, but only reported data for WAT, not BAT (Blaszkiewicz et al., 2019) - the darker tint of BAT preventing optimal tissue clearing and data acquisition. While sympathetic stimulation has been shown to be sufficient for increasing BAT thermogenesis, modern optogenetic and chemogenetic techniques have not been utilised to investigate the necessity of sympathetic drive for BAT thermogenesis (i.e. does inhibition of iBAT sympathetic drive ablate cold-induced thermogenesis?). Similarly, how metabolism in human BAT is centrally controlled remains unclear, and difficult to compare to animal models owing to divergence in tissue quantity.

**Figure 4:**
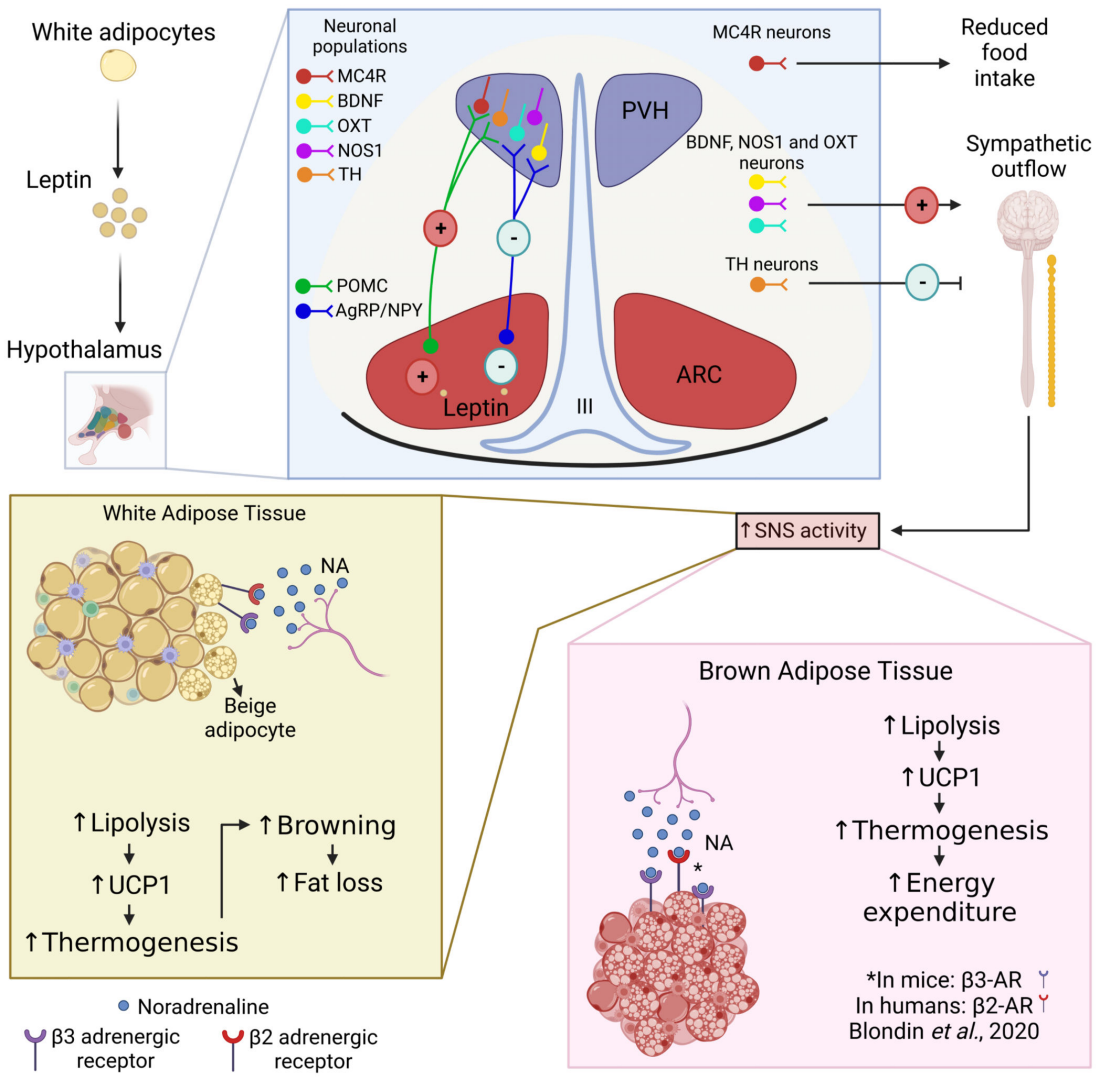
Pre-Sympathetic brain outputs to White and Brown Adipose Tissues. Central hypothalamic nuclei detect circulating leptin. Within arcuate (ARC), leptin activates anorexigenic pro-opiomelanocortin (POMC) neurons and inhibits orexigenic neuropeptide Y/agouti-related protein (NPY/AGRP) neurons and this increases sympathetic outflow to adipose tissues. Both populations project to different areas in the hypothalamus, especially to the paraventricular nucleus (PVH) that receives a dense input of these neurons. Melanocortin 4 receptor (MC4R), one of the key players in the regulation of energy expenditure, is densely expressed in the PVH where it has an essential role in regulating food intake (Garfield et al., 2015) but not directly in energy expenditure (Balthasar et al., 2005). However, MC4Rs in other sites rather than in the PVH, like the median preoptic nucleus, dorsomedial or sub zona incerta, seem to mediate melanocortin effects on the energy expenditure (Monge-Roffarello et al., 2014, Vaughan et al., 2011, Voss-Andreae et al., 2007). Non-MC4R-expressing neurons in PVH, like oxytocin (OXT) neurons or nitric oxide synthase-1 (Nos1) expressing neurons, can regulate thermogenesis increasing energy expenditure (Sutton et al., 2014). Projections from ARC NPY neurons to PVH tyrosine hydroxylase (TH)-expressing neurons are important to modulate energy expenditure by reducing sympathetic activity in brown adipose tissue (BAT) (Shi et al., 2013). Through brain-derived neurotropic factor-expressing (BDNF) neurons in the PVH, leptin exerts actions in the sympathetic innervation in the adipose tissue (Wang et al., 2020). In addition (and not represented in the figure), LepR+ dorsomedial hypothalamus (DMH) neurons regulate brown adipose tissue (BAT) thermogenesis and browning. Similarly, median preoptic nucleus (MPO) LepR+ and ventromedial nucleus (VMH) LepR+ neurons are also implicated in regulating both energy expenditure and thermogenesis (not represented). In BAT, sympathetic noradrenaline (NA) acts on β3-adrenoceptors (β2 in humans) to increase lipolysis and lipid metabolism. The mitochondrial H+ gradient established by substrate oxidation is then dissipated across the inner membrane via UCP-1, causing energy to be dissipated as heat rather than stored chemically. In white adipose tissue (WAT), sympathetic acts on β3-adrenoceptors to increase lipolysis, liberating FFAs to be metabolised elsewhere. In addition, adrenergic signalling can encourage a phenotype change, causing WAT to adapt for non-shivering thermogenesis, beige adipose tissue (BeAT). Like BAT, BeAT expresses UCP-1 to dissipate electrochemical energy as heat. All three mechanisms reduce fat mass, thus closing a homeostatic feedback loop of leptin action.

In contrast with the challenges observed in the study of sympathetic signalling to BAT, several reports have highlighted the hypothalamic origin of such networks (Figure 4). One of the first studies observed that leptin effects on decreasing body weight was partly because of its role in activating POMC/CART (pro-opiomelanocortin / cocaine and amphetamine-related transcript) neurons signalling to sympathetic preganglionic neurons in the thoracic spinal cord, and that this was related to increased thermogenesis (Elias et al., 1998). Reports that followed observed roles for adrenocorticotropic hormone (ACTH)-dependant stimulation of BAT (Fukuhara et al., 1996, Zaretskaia et al., 2002, Rothwell and Stock, 1985, Rothwell et al., 1991), which was in line with POMC neuron activity, as ACTH is one of the subproducts of POMC processing. Further investigation found that besides indirect adrenergic stimulation, POMC subproduct signalling had a role in thermogenesis through activation of the melanocortin receptor 4 (MC4R) in the CNS (Vaughan et al., 2011, Voss-Andreae et al., 2007, Brito et al., 2008, Tung et al., 2007), which led to increased sympathetic activity in the BAT (Chitravanshi et al., 2016). MC4R is densely expressed in the PVH where it has an essential role in regulating satiety (Garfield et al., 2015) but not directly in energy expenditure (Balthasar et al., 2005). Therefore, melanocortin’s effects on energy expenditure seem to be connected to other brain areas like the median preoptic nucleus, the dorsomedial or sub zona incerta (Monge-Roffarello et al., 2014, Vaughan et al., 2011, Voss-Andreae et al., 2007). Interestingly, MC4R in sympathetic cholinergic preganglionic neurons of the intermediolateral nucleus (IML) of the spinal cord have a critical role in melanocortin actions on energy expenditure, regulating the thermogenic response to diet and cold challenges, and also the *beiging* in WAT (Rossi et al., 2011, Berglund et al., 2014).

Indeed, tracing studies with PRV have determined three major components in polysynaptic circuits projecting from the hypothalamus to the BAT - i) premotor neurons expressing POMC/CART in the hypothalamus (mainly in the ARC and PVH, but also lateral anterior hypothalamus (LAH), DMH and VMH nuclei) and in the brainstem, ii) spinal cord and iii) stellate ganglion (Wiedmann et al., 2017). Additional studies suggest that AgRP/NPY (agouti-related peptide / neuropeptide Y) neurons in the ARC also have a role in controlling BAT sympathetic input and thermogenic output (Shi et al., 2013, Kuperman et al., 2016). Lastly, it was shown that leptin signalling in POMC and AgRP neurons also regulated BAT sympathetic innervation through a circuit of brain-derived neurotrophic factor (BDNF)- expressing neurons in the PVH (Wang et al., 2020). It is important to mention that BDNF is a key regulator of energy expenditure and is expressed in several brain regions, not being a specific marker in PVH. However, although it is largely still unknown the role of this factor in energy balance, the deletion of the *Bdnf* gene in other brain areas like the VMH and DMH only produces modest obesity (Unger et al., 2007). By contrast, the deletion of *Bdnf* in the PVH regulates food intake, drives thermogenesis and boosts sympathetic outflow (An et al., 2015), suggesting the PVH as a main site of action (but not exclusively).

Given the complexity of hypothalamic networks, the precise signalling involved in downstream activation of sympathetic activity still needs to be further understood - however, it should be noted that its investigation has been focused on central pathways and assumed noradrenergic signalling as the single effector mechanism responsible for the observed phenotypes, a major caveat also present in studies investigating sympathetic functions in other tissues.

Increasingly, bidirectional communication between BAT and SNS is implicated in the remodelling response. Selective BAT overexpression of adipocyte-secreted Bone Morphogenetic Protein 8b (BMP8b) in mice increases sympathetic innervation and BAT vascularisation, even in mice housed at warm temperatures (Pellegrinelli et al., 2018). Transcriptomic analysis of adipocyte-expressing sympathetic growth regulators showed that only neuregulin-4 *(Nrg4)* was significantly upregulated with BMP8b overexpression, and down-regulated in BMP8b knockouts. Exogenous application of NRG4 promoted sympathetic axon growth and branching, suggesting BAT could dynamically remodel surrounding tissue in response to BMP8b (Pellegrinelli et al., 2018). Similarly, calsyntenin-3β (CLSTN3ß) binds to and enhances the expression of neurotrophic factor S100b, which in turn encourages local sympathetic growth and arborisation (Zeng et al., 2019). CLSTN3ß silencing or upregulation reduces or enhances sympathetic innervation in BAT, respectively, and CLSTN3ß ablated subjects on a high fat diet were predisposed to develop obesity. Genetic silencing of S100b phenocopies CLSTN3ß deficiency, and exogenously driven S100b expression in BAT restores defective sympathetic innervation in the CLSTN3ß-deficient genotype (Zeng et al., 2019). Adipocyte-derived factors represent an important component in remodelling the BAT sympathetic supply to utilize more energy – a better understanding of this process could offer targets to help upregulate BAT activity.

Finally, emerging evidence suggests BAT is an integrated metabolic tissue with endocrine functions (Villarroya et al., 2017) and roles in glucose homeostasis (Li et al., 2021). The contribution of SNS to these non-thermogenic functions of BAT is poorly understood. In-vivo measurements of sympathetic neural signalling using genetically encoded markers of neuron activity are yet to be performed but understanding how homeostatic information is encoded by sympathetic signals could help delineate the contributions of the SNS to thermogenic and diverse non-thermogenic functions of BAT. Given the known impairment of adequate thermogenesis in obesity (Levin et al., 1981, Jung et al., 1979, Carey et al., 2013) and its potential as a therapeutic target for weight-loss independently of reduction of food intake, further investigation of how precise sympathetic circuits regulate its functions is desired.

### White Adipose Tissue

Noradrenergic signalling has long been known to cause WAT lipolysis. Challenges presented by imaging adipose tissues and manipulating its function have meant that an understanding of how NA is supplied and regulated within WAT has been elusive until recently. Early imaging studies suggested that WAT received some innervation (Boeke, 1933, Hausberger, 1934) but the silver and platinum-based stains employed lacked the required tissue specificity to delineate neuron identity. WAT was not investigated in early fluorescence microscopy studies regarding sympathetic innervation of adipose tissue and has proven difficult to image using conventional histological techniques (Wirsen, 1964). The relatively sparse nature of innervation means that sectioning adipose samples can result in neurons being presented as puncta, making structural analysis of adipose innervation difficult. In addition, lipids scatter light and vascular networks within the tissue often auto-fluoresce, introducing artifacts and hampering image resolution. As a result, until recently, it was unclear if white adipocytes received direct synaptic innervation from SNS or if catecholaminergic signalling was perivascular in nature.

Only with recent advances in two-photon imaging and tissue clearing it has been possible to visualise entire sympathetic arborizations as well as confirm the presence of neuro-adipose junctions. In 2015, Zeng and colleagues used optical projection tomography approach to reconstruct 3D images of entire inguinal fat pads. Two photon microscopy of catecholaminergic reporter mice labelled with lipophilic dye showed that 50% of all neurons innervating fat pads were sympathetic, and bouton-like structures innervated 8% of adipocytes. Advanced tissue clearing preparations have allowed for direct imaging of entire fat pads (Zeng et al., 2015). In 2017, using these techniques, Jiang et al reported that 98.8% of presynaptic (synaptophysin-positive) fibres within WAT were sympathetic (TH-positive), and these neurons were in ‘close apposition’ with 91.3% of adipocytes, although the authors do not provide a numerical criteria of how apposition was defined (Jiang et al., 2017). In both studies above for example, the authors quantify neuron- adipose apposition through analysis of finite ‘representative’ areas and in doing so cannot describe the regional anatomy of the fat pad - which could account for the large discrepancy of the numbers mentioned earlier. Development of artificial intelligence (AI) and Machine Learning techniques to conduct image processing of the entire fat pad should soon be a reality. In addition, fat pads are by their nature thick and thus homogenous staining of the entire tissue might be difficult to achieve. Finally, clearing media can reduce the lifespan of fluorophores and some require the tissue to be constantly immersed during imaging, meaning specialised objective lenses are needed to solve refractive index matching. Willows and colleagues developed a new technique that mechanically compresses the adipose depot, reducing tissue thickness. This method makes whole adipose-organ imaging more widely accessible, simplifying tissue processing and permitting imaging with more affordable and common widefield epifluorescence and laser scanning confocal microscopes. However, this method does struggle to achieve the same neurite resolution and analysis of nerve distributions in the compressed z-axis cannot be reliably performed due to the mechanical manipulation of the tissue (Willows et al., 2021). Still, such higher throughput, more accessible imaging methodologies will be useful to probe WAT-SNS structure and function in greater depth.

More macroscopically, retrograde tracing techniques have aimed to map the neuroanatomical origin of pre- and post-ganglionic fibres innervating WAT and suggest that distinct fat pads receive innervation from distinct populations of neurons. Early fluorogold retrograde tracing studies in Siberian Hamsters claimed dense cell bodies projecting to inguinal WAT (iWAT) from T13 abdominal sympathetic ganglion, although the study did not provide representative micrographs of ganglia (Youngstrom and Bartness, 1995). More modern PRV transsynaptic retrograde methods echo these findings, indicating significant iWAT innervation from in T13/L1 sympathetic ganglia, although the transsynaptic nature of the reporter overshadows the claim of a direct mono-synaptic connection (Wiedmann et al., 2017) (Figure 3). The use of transgenic mouse models offers better precision in neuronal tracing still, Huesing and colleagues injected PRV-GFP into iWAT of sympathetic TH-tomato reporter mice and imaged whole cleared torsos containing spinal cord and paravertebral ganglia to identify coexpression and thus the preganglionic and postganglionic inputs to iWAT. They found postganglionic innervation from sympathetic ganglia T12-L1 and preganglionic inputs from IML of T7- T10, although the authors also reported a minority of subjects with labelled preganglionic neurons from T5-T12, and postganglionic neurons from T7-L2 (Huesing et al., 2020) (Figure 3). Interestingly, the authors reported sympathetic fibres innervated ‘dorsolateral’ iWAT via lateral cutaneous rami of intercostal nerves, and ‘inguinal’ WAT via the anterior cutaneous branch of the femoral nerve – predominantly somatic nerves and declared exclusively somatic by many leading neuroanatomical atlases. The relatively superficial position of these mixed nerves with lipolytic function could be an attractive target for percutaneous neuromodulatory anti-obesity treatments. In addition, the authors highlight that inferences from macroscopic dissection studies do not always hold, and give imperative to molecularly analyse the sympathetic neuroanatomy of other tissues and organs.

While most neuroanatomical studies tend to present harmonious findings, a 2017 study received a lot of attention and offered a different framework of iWAT neuroanatomy. The authors used an Alexa-dye-conjugated Cholera Toxin Subunit B (CTB) injection into iWAT and reported labelled neurons almost exclusively in the coeliac ganglia – a structure providing visceral sympathetic innervation to kidney, liver and GI organs. The authors reported very few cells innervating iWAT originating from either the spinal cord or lumbar dorsal root ganglia (Jiang et al., 2017). These results have not yet been reproduced, however – a study attempting to do so found only 25% of subjects reported PRV+ Coeliac Ganglia (Huesing et al., 2020). The large injection volumes and long incubation times employed by Jiang *et al.* likely caused tracer to diffuse via the inguinal canal to infect abdominal organs, highlighting a common pitfall in retrograde viral tracing studies.

Some deeper adipose pads also appear to be supplied by post-ganglionic neurons that synapse not in the paravertebral sympathetic chain but in mid-line autonomic ganglia. Using a green fluorescent protein labelled adeno-associated retrograde virus tracing technique, Cardoso et al observed cell bodies of the efferent sympathetic nerves innervating gonadal WAT (gWAT) in the aorticorenal ganglion (Cardoso et al., 2021). In addition, these authors defined the higher circuits implicated in this new gWAT-aorticorenal axis, observing polysynaptic connections to overlapping but distinct brain areas, associated with SNS, especially the PVH (Cardoso et al., 2021). SNS innervation to WAT is complex, varied, and tissue dependent – only in the last few years has appreciation of its detail reliably emerged.

Indirect evidence of sympathetic-induced lipolysis has existed for over 60 years - plasma free fatty acid concentrations were noted to increase with noradrenaline administration in rats and with electrical stimulation of adipose nerves in dogs, and these changes were inhibited by trimethaphan camphorsulfonate ganglionic blockade and peripheral ß-adrenergic antagonism (Bogdonoff et al., 1960, Steinberg et al., 1961, Rosell, 1966, Goodner et al., 1967, Fredholm and Rosell, 1968). The anatomical challenges of accessing sympathetic tissue, tissue-specificity and use of methodologies that rely on tissue slicing has made specific, direct evidence of SNS-induced lipolysis activity in vivo historically difficult to achieve. Mechanical denervation and electrical stimulation approaches lack tissue specificity as WAT also receives innervation from non-sympathetic neurons (Zeng et al., 2015) (Wang et al., 2022). For example, sensory and sympathetic fibres travel together in bundles so surgical denervation cannot distinguish between them (Wang et al., 2022). Systemic administration of adrenergic receptor agonists and antagonists impacts other tissues that express the various forms of adrenoreceptors and does not quite mimic the localized delivery that is achieved by tissue-resident axons that can also be subject to potential circuity dynamics – within ganglia, or across them. Classic ‘Noradrenaline Turnover’ (NATO) approaches that require systemic blockade, specimen culling and tissue homogenisation lack specificity and fail to capture signalling dynamics (Young et al., 1982, Garofalo, 1996, Migliorini et al., 1997, Nguyen et al., 2014).

Direct evidence that sympathetic activation was sufficient to drive lipolysis has only emerged since the advent optogenetic and chemogenetic techniques that can stimulate or inhibit the activity of molecularly defined neuronal populations. By crossing TH-Cre mice to a Cre-dependent ChR2 line, Zeng et al showed optogenetic activation of tissue specific sympathetic neurons released NA (as measured by ELISA assay) and chronic stimulation depleted fat mass in illuminated areas (Zeng et al., 2015). Similar results were observed when leptin was administered, causing local increases in NA and reduced fat mass. Local genetic ablation of sympathetic adipose neurons or local knockout of dopamine ß-hydroxylase (a key NA synthetic enzyme) blocked leptin induced lipolysis. Thus, the authors presented the first functional evidence that specific adipose sympathetic signalling is sufficient to induce lipolysis and necessary for leptin-induced lipolysis to occur (Zeng et al., 2015). Similarly, Wang et al showed specific deletion of LepR+ neurons in ARC (but not other hypothalamic nuclei) resulted in significant decreases the sympathetic innervation to mouse iWAT and BAT (Wang et al., 2020). However, understanding the dynamics of sympathetic or noradrenergic signalling in response to pro-lipolytic stimuli remains elusive. Wang et al. imaged immunolabelled TH+ fat pads to demonstrate structural loss of innervation, while Zeng et al approximated, NA activity via ELISA assay after surgical removal of the fat pad. Thus, neither study directly described how noradrenergic signalling changes in response to central pro-lipolytic signals. To date, studies measuring sympathetic activity in vivo and in real time using markers of neuronal activity, e.g. GcaMP6, or measuring NA activity, e.g. via fast-scan cyclic voltammetry (Ishida et al., 2018) are yet to be performed, largely as achieving a stable mount to image the adipose tissue has proven difficult. Such studies could provide valuable insights into the signalling dynamics controlling lipolysis.

Similarly, the functional spatial control of SNS signalling is poorly understood. The apparatus for spatially restricted control of lipolysis is present – WAT is innervated from discrete spinal levels and sympathetic ganglia and local SNS-WAT communication is performed at the Neuron-Adipose Junction. Functional evidence based on limited NATO methods do support the notion of some regional specialisation in lipolysis. In response to a 16 hour fasting challenge, sympathetic outflow is significantly higher in iWAT over epididymal WAT (eWAT) (Nguyen et al., 2014), while in response to cold exposure, NA turnover increased in iWAT and eWAT, but not in retroperitoneal WAT (rWAT) (Brito et al., 2008). SNS signalling also appears to be temporally dynamic - at the start of a 12 days of a restrictive calorie diet, eWAT increased early on, but by day 12 sympathetic activity towards iWAT had increased and indeed predominated (Brito et al., 2008). The significance of regional selectivity in lipolysis is unclear, but it could explain the sexual dimorphism that is known in humans: women tend to accumulate fat subcutaneously whereas man do so viscerally. Still, understanding the anatomy of WAT innervation is important for experimental design, and may allow for therapeutically intervention via non-invasive spatially restricted modulation of neuronal activity (Stanley et al., 2012, Stanley et al., 2015, Rossini et al., 2015, Rabut et al., 2020).

Finally, ambiguity still surrounds the morphological changes to sympathetic axons during cold and fasting challenges – namely, whether sympathetic arborisations change their density or localisation to facilitate lipolysis. Earlier studies suggested an increase in neuronal arborisations and increases in TH+ neuron density in WAT after cold acclimatization but these observations were based on analysis of tissue sections that poorly represent the entire fat pad (Murano et al., 2009, Vitali et al., 2012). Whole tissue clearing methods have allowed for more reliable quantification of neuronal structures within adipose tissue, but results are conflicting. Two studies supported increases in neural arborisation in response to cold challenges. First, Blaszkiewicz et al in 2019 reported increased neural arborisation in iWAT following a cold challenge, especially around the around the local lymph nodes (Blaszkiewicz et al., 2019). Second, Jiang et al also found increases, but the authors did not quantify or distinguish the anatomical areas examined (Jiang et al., 2017). Conversely, Chi and colleagues reported no increase in cold-induced nerve density in iWAT, but did report statistically significant increases in TH protein expression, suggesting increased SNS density or upregulation of synaptic function (Chi et al., 2018). The different conclusions of these two reports could result from methodological differences. While both studies performed advanced sub-anatomical quantification of similarly aged mice of the same strain,

Blaszkeiwicz used a 10-day cold challenge and analysed 25mm^2^ of iWAT per sample, while Chi’s protocol lasted only for 7 days and analysed ten cubic ‘representative’ areas of unspecified size. In addition, Blaszkeiwicz used the pan-neuronal marker PGP9.5 for neuronal quantification, whereas Chi used TH, specific for sympathetic neurons. Recently, Willows and colleagues quantified neurite densities across entire adipose tissues using mechanical compression method, and found a non-significant increase in neurite densities in cold adapted mice, but only examined four mice in a proof-of-concept study (Willows et al., 2021). Of note, in none of the described studies were the structural changes associated with the cold/fasting challenges the primary focus – a study combining and assessing the above methodologies to give a more detailed description of SNS-WAT innervation and adaptations in health would be relevant, especially considering the role of adipose tissue innervation for whole body metabolism.

### Beige Adipose Tissue, BeAT

In response to a cold challenge, WAT can undergo a process of significant structural and biochemical remodelling, referred to as browning or *‘beiging’* of WAT. This process generates a phenotypically intermediate tissue between WAT and BAT, often termed Beige Adipose Tissue (BeAT) that appears to play important roles in the regulation of metabolism. Because BeAT exhibits increased mitochondriogenesis and expresses a significant amount of UCP-1, it is thought that it has the capacity to generate heat through uncoupling of the electron transport (Harms and Seale, 2013, Zhu et al., 2016). However, PET-CT experiments have shown that BeAT, even in the absence of functional BAT, is very weak at enhancing its oxidative activity, thus its ability to participate in non-shivering thermogenesis has been questioned (Labbé et al., 2016, Labbé et al., 2018). Nevertheless, BeAT definitely has the ability to participate in systemic substrate metabolism through the uptake of glucose, and the sympathetic activity is central in initiating and controlling this process. Chronic β-adrenergic agonism induces browning in WAT (Cousin et al., 1992) and genetic or pharmacological ablation of adipose sympathetic supply stops cold-induced browning (Jiang et al., 2017, Cero et al., 2020). In addition, sympathetic neurons innervate BeAT-inducible tissues (e.g. iWAT) at a higher density than classic white (e.g. eWAT) and browning occurs in subcutaneous fat pads in areas with the highest density of amount of sympathetic innervation (Chi et al., 2018).

Sympathetic signalling potentiates BeAT activation and differentiation. In mice, neonatal BeAT prevalence, as measured by expression of UCP1+ expression, peaks at day 20 before gradually reducing (Xue et al., 2007). This reduction is associated with diminished TH+ expression in immunoblotting sections (Xue et al., 2007) and reduced nerve fibre density, length and branching points as assessed with tissue cleared 3D imaging (Cui et al., 2021). Similarly, iWAT β-adrenergic receptor knockouts maintained sympathetic arborisation density and activation in response to cold challenge, but had reduced appearance of multilocular BeAT and no increase in *beiging*-associated genes (Chi et al., 2018), suggesting that β-adrenoceptor activation is necessary for BeAT trans-differentiation.

While browning has been shown to occur in humans, this has only been confirmed in states of severe and systemic adrenergic stress – namely major burn trauma (Patsouris et al., 2015, Sidossis et al., 2015), cancer-associated cachexia (Petruzzelli et al., 2014) and pheochromocytoma (Frontini et al., 2013). Understanding the mechanisms initiating browning locally have received emerging attention as a method to treat metabolic syndromes by physiologically increasing the capacity for energy expenditure, often improving insulin resistance (Fang et al., 2015) and encouraging metabolic reprogramming (Barquissau et al., 2016). There are significant gaps in understanding how transcriptional programmes confer these changes but are being investigated as useful targets to initiate *beiging* for therapeutic gain.

The transcriptional regulators controlling BeAT trans-differentiation are only partially understood, but sympathetic-induced browning signalling is, in part, conducted and amplified via gap junctions, in particular Connexin-43. In 2017, the Scherer lab performed transcriptomic analysis of WAT tissue following a cold challenge and found increased Gja1 (Cx43 gene) expression which could be replicated by β3 adrenoceptor agonism (Zhu et al., 2016). UCP-1 promoter driven Cx43 deletion had no effect on the thermal responsivity and physiology of BAT, but blunted cold-induced WAT metabolic changes to a similar degree as adipose tissue denervation. Similarly, pharmacological blockade of Cx43 inhibited *beiging* in response to central sympathetic activation and WAT specific Cx43 overexpression promoted *beiging* at room temperature. Thus, increased intracellular [cAMP] resulting from cold-induced sympathetic activity appears to upregulate expression of key BeAT proteins and Cx43 expression and assembly. This, in turn, enhances diffusion of *beiging*/thermogenic signalling to adjacent adipocytes, even those not directly innervated by NAJ, initiating clusters of white adipocytes to undergo *beiging* (Zhu et al., 2016).

The understanding of downstream BeAT transcriptional regulators is incomplete but is not entirely absent. For example, upregulation of PR domain-containing protein-16 (PRDM16), a transcriptional regulator, is sufficient to initiate differentiation of beige adipocytes in subcutaneous WAT (Seale et al., 2011, Nagano et al., 2015), and improve glucose tolerance and energy expenditure in response to a high-fat diet challenge (Seale et al., 2011). Conversely, adipocyte-specific deletion of PRDM16 inhibits beige adipocyte formation and function, increasing the propensity for mice to develop dietary obesity, insulin resistance and hepatic steatosis (Cohen et al., 2014). In addition, PRDM16 exerts important sympathetic effects to remodel local sympathetic neurons and facilitate *beiging* and thermogenesis. 3D imaging of tissue cleared iWAT shows adipocyte-specific PRDM16 knockouts are unable to keep the regional differences in SNS density within the different sub-regions in iWAT, whereas the WT control showed a notable regional difference in medial vs lateral sub-regions of iWAT (Chi et al., 2018); this results showed the importance of PRDM16 regulating regional neurite projections. 3D imaging of tissue cleared iWAT an adipose-specific inducible PRDM16 knockout showed silencing of PRDM16 during post-natal days 6-28 caused a reduction in early post-natal beige adipocytes and sympathetic neurite density (Chi et al., 2021). Bidirectional communication between BeAT and sympathetic neurons is important in the facilitation and propagation of browning.

## Neuroendocrine Loop of Metabolic Control

**Figure 5:**
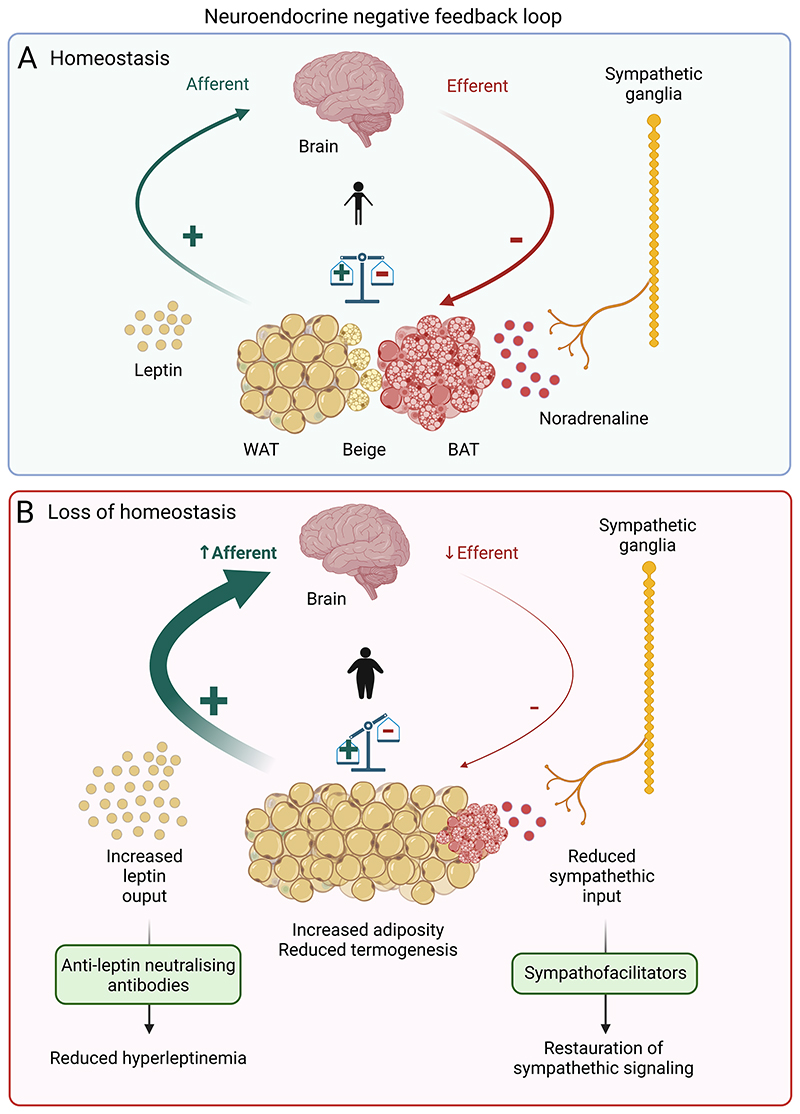
Working models for reversing loss of homeostasis of the neuroendocrine loop of leptin action. Leptin is produced by the adipose tissue and exerts its main actions through the brain, mainly in the hypothalamus’ arcuate and indirectly in the paraventricular nuclei. In health, increased leptin results in an increase in sympathetic signalling to the adipose tissues, increasing lipolysis and thus reducing plasma leptin. In chronic metabolic disease, persistently elevated leptin can cause insensitivity to signal, so-called ‘Leptin Resistance, which is concomitant with a decrease in sympathetic tone onto the adipose tissues, ultimately leading to sympathetic neuropathy. Together, the afferent and efferent arms of the metabolic homeostatic feedback loop are impeded, and a state of metabolic dysregulation emerges, causing obesity and other metabolic syndromes. Sympathofacilitators aim to selectively supplement peripheral sympathetic signalling and encourage lipolysis. Hyperleptinemia driving leptin resistance can be reduced with anti-leptin neutralising antibodies, allowing sensitisation to leptin. In tandem, the neuroendocrine negative feedback loop governing metabolism can be restored back to homeostasis.

Homeostatic neuroendocrine feedback loops maintain homeostasis via afferent (sensory) signals that are integrated and processed centrally to generate efferent (motor) outputs. In the context of metabolism, leptin is the primary afferent signal, information regarding metabolic state is computed primarily in the hypothalamus, and the SNS signalling acts as the effector arm (Figure 5).

Leptin is an adipokine synthesised proportionally to adipose tissue mass. Global increases in fat mass raise plasma leptin concentrations to increase energy expenditure and reduce food intake (Frederich et al., 1995, Rosenbaum et al., 1996). Conversely, decreases in global adipose mass and congruent reductions in plasma leptin decrease energy expenditure and increase food intake. In mice and humans lacking a functional leptin gene plasma leptin is undetectable and an obese phenotype is observed that is readily reversed by leptin administration (Zhang et al., 1994, Farooqi et al., 1999). Similarly, exogenous administration of leptin significantly decreases body weight and adipose tissue mass without affecting lean mass in wild-type mice, but has no effect in mice lacking the leptin receptor (Campfield et al., 1995, Halaas et al., 1995, Maffei et al., 1995, Tartaglia et al., 1995).

Immediately following leptin’s discovery (Zhang et al., 1994), the prevailing belief was that it solely controlled energy balance by reducing appetite and thus inducing hypophagia. This notion came from studies showing leptin administration reduces food intake (Campfield et al., 1995, Pelleymounter et al., 1995, Weigle et al., 1995). Leptin directly activates anorexigenic POMC neurons and inhibits orexigenic NPY/AGRP neurons (Stephens et al., 1995, Cowley et al., 2001) while also modulating higher centres conveying satiety and the reward value of food (Farooqi et al., 2007, Domingos et al., 2011, Atasoy et al., 2012). However, this is an incomplete description of leptin’s control of metabolism. Pair-feeding experiments show the weight loss observed post-leptin administration cannot, in the long term, be accounted for by decreased food intake alone. When leptin deficient (ob/ob) mice were fed the same amount of food as leptin-treated ob/ob mice, they dropped weight similarly in the first week of treatment, but diverged afterwards as they had less reduction in body and fat-depot weights (Levin et al., 1996). Similarly, when pair fed and kept at thermoneutral conditions, a 15-day course of leptin treatment in ob/ob mice resulted in energy expenditure twice as great as in pair-fed controls (Rafael and Herling, 2000). Thus, leptin acts not just to satiate, but to directly regulate energy expenditure.

Sympathetic signalling is an important mechanism by which leptin increases energy expenditure. Early studies noted that chronic β3-adrenergic agonist treatment mimicked leptin application, reducing adipose tissue mass and leptin expression while sparing lean tissue mass in mice (Collins et al., 1996). Some mused that adrenal medulla could be the source of catecholaminergic signalling, but as adrenalectomized ob/ob mice showed no difference in body weight reduction and adipose reduction compared to sham-operated ob/ob controls, the lipolytic action of leptin was thus independent of the adrenal gland (Arvaniti et al., 1998, Gemmill et al., 2003).

By deduction, SNS signalling was the most likely mediator of leptin’s effects on energy expenditure. This was bolstered by studies showing intravenous (IV) infusion or intra-hypothalamic delivery of leptin increases nerve activity to BAT. These measurements came from extracellular electrodes implanted over local neurovascular peduncles, so the activity of non-sympathetic neurons was also measured (Haynes et al., 1997, Rahmouni and Morgan, 2007). Similarly, some studies report that intraperitoneal leptin administration increases NATO in mouse iBAT but acquired their data by systemically administering alpha-methyl-p-tyrosine (AMPT) and measuring NA in homogenised post-mortem tissues, thus inhibiting all central monoaminergic pathways before making measurements (Collins et al., 1996). Structurally, sympathetic signalling and leptin activity are also closely related - specific deletion of key LepR+ neurons in ARC, pre-sympathetic BDNF-expressing neurons in PVH and LepR mutated ob/ob mice all result in reduced sympathetic innervation of adipose tissue (Wang et al., 2020).

Leptin crosses the blood–brain barrier (BBB) to bind to Leptin receptors (LepR), predominantly in the ARC, VMH, DMH and medial preoptic nuclei (MPO) (reviewed in Maffei *et al.* (Maffei and Giordano, 2021) , outlined in Figure 4) to increase sympathetic tone, increase energy expenditure and reduce food intake. Immunohistochemical and genetically encoded fluorescent reporters show LepR primary expressed in ARC, DMH, MPO. In addition, ARC LepR project to other hypothalamic areas for regulation of energy expenditure, (e.g. BNDF-expressing neurons in PVH (Wang et al., 2020)) and lateral hypothalamic areas to regulate mesolimbic dopaminergic motivation/reward systems. LepR+ DMH neurons regulate BAT thermogenesis and browning and show densely connections to pre-sympathetic BAT circuits (Zhang et al., 2011). Functionally, LepR+ DMH neurons are activated after cold exposure (Rezai-Zadeh et al., 2014). Also, chemogenetic activation of LepR+ DMH neurons is sufficient to stimulate BAT thermogenesis and energy expenditure, in a β3-adrenoceptor dependent fashion (Rezai-Zadeh et al., 2014, Enriori et al., 2011). In addition, genetic knockdown of NPY in DMH neurons increases browning specifically in subcutaneous WAT and this process is dependent of SNS innervation (Chao et al., 2011). Interestingly, there are two very different classes of DMH-LepR neurons: those in the ventral DMH, GABAergic neurons more related to the regulation of food intake (Garfield et al., 2016, Berrios et al., 2021), and those in the dorsal DMH, glutamatergic neurons involved in the regulation of energy expenditure. These last ones, marked by the expression of Bombesin-like receptor 3 ((Brs3)-expressing neurons), increase body temperature and BAT activity (Piñol et al., 2018). Specifically, the authors observed that specific optogenetic stimulation of DMH-Brs3 projections to the raphe pallidus (RPa) (a key sympathetic premotor site to control thermogenesis (Morrison et al., 1999, Morrison, 2003)), regulates body temperature, energy expenditure and heart rate.

MPO LepR+ neurons have recently been implicated in regulating both energy expenditure and thermogenesis in response to internal energy state (Yu et al., 2018). Similarly, VMH LepR+ show a selective increase to leptin administration in the Nucleus Solitarius and selective VMH LepR+ loss of function experiments in mice show that energy balance can be maintained in basal conditions, but not during positive energy balances (Seamon et al., 2019). An understanding of the downstream mechanisms of both is lacking.

The advent of techniques that allow tissue-specific control of neurons has yielded direct evidence of the necessity of SNS function to elicit leptin-induced energy expenditure. In 2015, Zeng and colleagues found tissue-specific genetic ablation of sympathetic neurons within a fat depot inhibited the lipolytic effect of leptin (Zeng et al., 2015). Similarly, in 2016, Pereira and colleagues used modified, BBB-impermeable diphtheria toxin to genetically sympathectomies a TH-Cre; LSL-DTR mouse strain and found doing so inhibited reductions in body weight in response to a leptin challenge and predisposed subjects to obesity upon a high fat diet challenge (Pereira et al., 2017). Both studies confirm that SNS signalling is a vital efferent mechanism to regulate metabolism. In addition, they highlight the inadequacy of the adrenal gland’s global noradrenergic discharge to describe the metabolic function of the sympathetic nervous system. Rather, SNS metabolic signalling aligns more tightly with the classical neuron doctrine and requires investigation accordingly.

Leptin resistance – a blunted response to leptin signalling, a weakened effector arm of this neuroendocrine homeostatic feedback loop – has emerged as an important pathological change in the development of obesity (Figure 5). This state emerges as chronic hyperleptinemia renders unable to meaningfully respond to disturbances in energy balance. Indeed, hyperleptinemia is required for the development of leptin resistance (Knight et al., 2010) and reducing leptin levels with neutralizing antibodies has been proven to restore homeostasis (Zhao et al., 2019). Understanding how leptin resistance develops and identifying mechanisms by which sympathetic effector signalling is disrupted has been central in attempts to rationally design effective antiobesity therapeutics.

### Insulin, another afferent signal

Insulin resistance and hyperinsulinemia are associated with obesity and are connected with hypertension problems in obese subjects. However, similarly to leptin resistance, the mechanisms involved in the dysregulation of this neuroendocrine loop are still under study. Insulin resistance and hyperinsulinemia are associated with obesity and are associated with hypertension problems in obese subjects. However, similarly to leptin resistance, the mechanisms involved in the dysregulation of this neuroendocrine loop are still under study.

Central insulin signalling is another important afferent signal in the metabolic feedback loop. There is accumulating evidence that insulin crosses the BBB into the brain and regulates different peripheral tissues and systemic metabolism (glucose production, lipolysis, lipogenesis, etc) and deletion of insulin receptors in the brain increases food intake and promotes obesity (Bruning et al., 2000). ICV insulin administration increases the sympathetic activity to the BAT, kidney, adrenal gland and hindlimb without modifying plasma insulin levels, and MAPK and PI3K seem to have a key role in this sympathetic outflow (Rahmouni et al., 2004).

However, other studies have shown the opposite. Insulin action in the brain is a critical regulator of WAT metabolism with inhibitory effects on sympathetic output. Thus, in lean rats, the sympathetic outflow to fat tissue is reduced and lipolysis suppressed after central insulin administration; but, like an inverse regulation, mediobasal hypothalamus insulin increases de novo lipogenesis in adipose tissue (Scherer et al., 2011). Nevertheless, the neuronal subtypes within the CNS involved on these actions was not clear.

Studies with neuron-specific insulin receptor knockout models have postulated some neuron populations as key players integrating the insulin signal. Thus, insulin signalling in POMC and NPY/AgRP neurons has an important role in regulating energy balance (Hill et al., 2010, Dodd et al., 2015, Dodd et al., 2017). Insulin receptor signalling in AgRP neurons seems to be essential in the control of WAT browning and energy expenditure (Dodd et al., 2017). Furthermore, the co-infusion of leptin and insulin results in an increase in TH staining, inguinal WAT browning and energy expenditure (Dodd et al., 2015), showing that both molecules can act synergistically in the CNS. Specifically, leptin/insulin infusion activates ARC POMC neurons, and this promotes an increase in iWAT browning and decreasing of adiposity through the SNS (Dodd et al., 2015). In addition, genetic alteration of the insulin receptor in these neurons has consequences on peripheral metabolism. Thus, specific deletion of insulin receptor in POMC neurons impairs insulin actions in adipose tissue lipolysis (suppression of lipolysis) but does not have consequences in its role in the glucose hepatic production in the liver. By contrast, after insulin receptor deletion in AgRP neurons, the capacity of insulin to suppress lipolysis was intact, however, the suppression of glucose hepatic production was damaged (Shin et al., 2017).

## Sympathetic Neuropathy and Metabolic Syndrome

Sympathetic innervation of metabolic tissues is essential for maintaining metabolic health, but metabolic dysregulation increase the propensity for neuropathy within these tissues. Such an autonomic neuropathy may trigger or exacerbate metabolic diseases, initiating a positive feedback cycle seemingly difficult to break.

Peripheral sensorimotor neuropathies are common in patients with metabolic syndromes, especially diabetes. While obese adipose tissue takes on a fibrotic quality that is difficult to image (Willows et al., 2021), 3D imaging of tissue cleared adipose tissue has shown that sympathetic neuropathic changes develop early in ob/ob mice (Jiang et al., 2017, Willows et al., 2021). Murine fat samples imaged at 24 weeks old show reduced sympathetic innervation and loss of synaptic markers (PSD95, GAP43) in iWAT. In iBAT, despite compensatory increases in synaptic markers and neurotrophins, overall sympathetic innervation was reduced, and the tissue had started to undergo WAT-like phenotypic changes - increased lipid accumulation and reducing expression of thermogenesis markers (UCP1, Cidea, Dio2), and a lightening in colour (“whitening”). Similar sympathetic neuropathic changes have been reported in human tissues – Blaszkiewicz et al reported reduced TH expression in the iWAT of obese and aged subjects (Blaszkiewicz et al., 2019).

Similarly, hepatic sympathetic neuropathy has been reported during diet-induced obesity (DIO) or in leptin deficiency (Liu et al., 2021). Liu and colleagues observed a retraction of the sympathetic nerves innervating the liver in obese models, these changes were prevented by calorie restriction or leptin replacement. These results are in contrast with a previous study reporting increased activity of hepatic sympathetic nerves in DIO (Hurr et al., 2019). However, it should be noted that electrophysiological measurements were conducted on the nerve segments outside of the liver lobes. Moreover, animals were fed the high-fat regimen for only 10 weeks (Hurr et al., 2019), and at least 20 weeks were necessary for Liu *et al* to observed liver neuropathy (Liu et al., 2021). In alignment with this, Silva and Caron propose a bimodal model of autonomic pathology, whereby an initial compensatory increase in hepatic sympathetic signalling is followed by a progressive sympathetic neuropathy that potentiates the development of metabolic disease (Silva and Caron, 2021). In this hypothesis, hepatic sympathetic dysfunction varies with time, thus assessing hepatic function and morphology in conjunction with hepatic sympathetic supply is important for studies that aim to establish the integrity of hepatic metabolic signalling. In addition, Silva and Caron suggest that prevention of early compensatory increases in hepatic sympathetic signalling and a reduction in the inflammatory and oxidative processes that contribute to neuropathy are potential targets to help halt sympathetic degeneration (Silva and Caron, 2021).

## Neuroimmunometabolism

Understanding the mechanisms by which SNS signalling to metabolic tissues is altered in disease states remains an important question in designing effective therapeutics. In recent years, the interactions between SNS, metabolic organs and local immune cells have emerged as a key contributor to pathological signalling changes. The central role of these interactions have led to the development of the growing field of neuroimmunometabolism, which investigates how these systems are interconnected and how these connections can be explored for therapeutic interventions.

The presence of the relevant apparatus for SNS signalling, immune cells and adipokines to interact has been known for some time. All primary and secondary immune organs are innervated by postganglionic sympathetic nerves (Bulloch and Pomerantz, 1984, Felten et al., 1985) and both innate and adaptive immune cells express adrenergic receptors. Similarly, leptin receptors are expressed on monocytes, neutrophils, eosinophils, basophils, dendritic cells, and NK cells and can regulate immune cell activation, differentiation, survival and chemotaxis (Larabee et al., 2020). Also, insulin receptors are also expressed in immune cells, where can modulate cell differentiation and polarisation (van Niekerk et al., 2020). Recent functional interrogations have highlighted how all three interact to regulate metabolism in health, and in disease.

### Neuroimmunometabolism in Pancreatic Tissues

**Figure 6:**
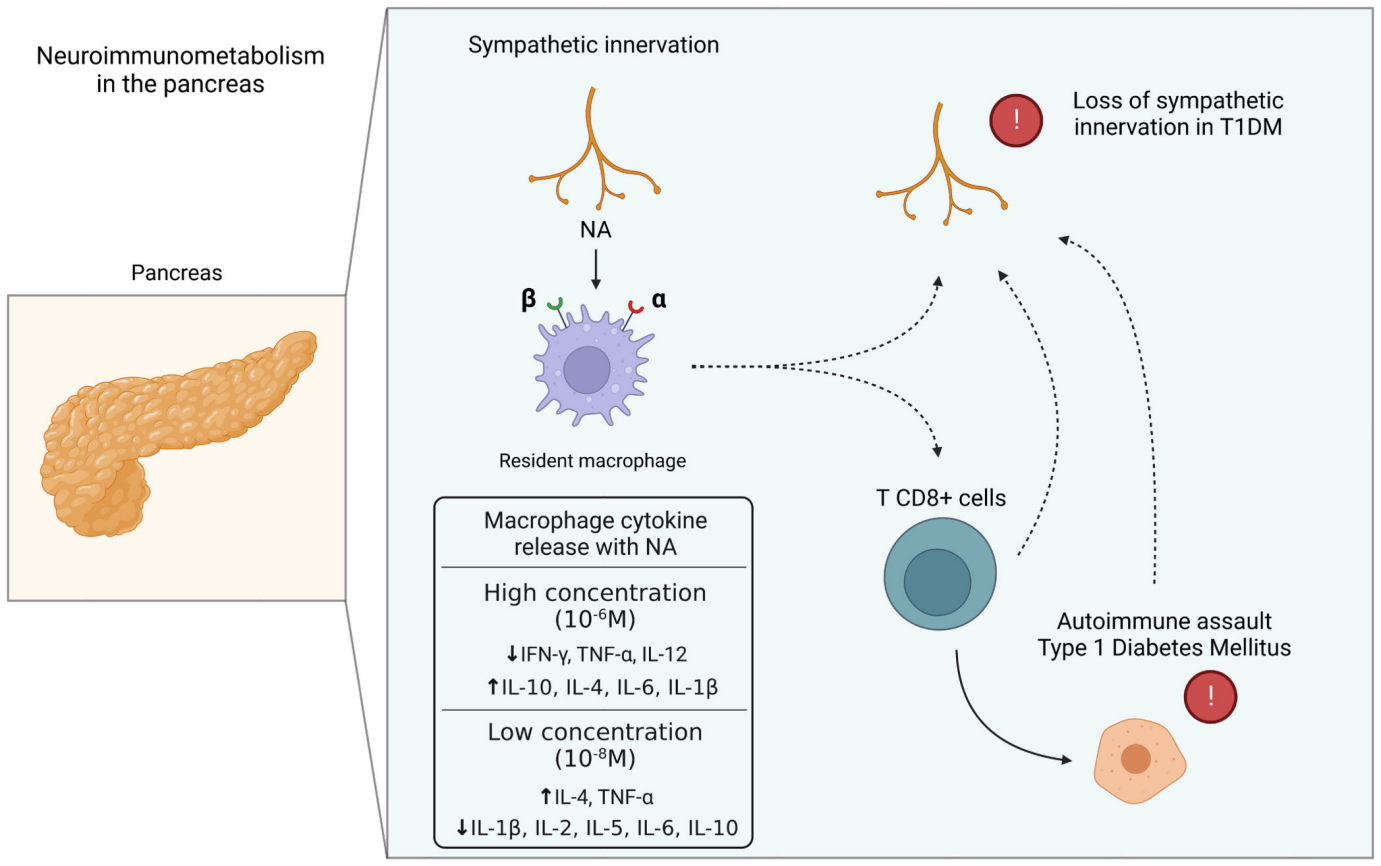
Neuroimmunometabolism in the pancreas. Noradrenaline (NA) is released by sympathetic nerve terminals within the pancreas. RNA sequencing of isolated islet macrophages incubated with high (10^-6^ M) or low (10^-8^ M) concentrations of NA indicates different patterns of cytokine activation. Treatment with high concentrations, assumed to mimic the concentrations experienced near nerve terminals, has anti-inflammatory effects via activation of low affinity *β2* adrenergic receptors. High concentrations of NA reduced the expression of INF-γ, TNF-α and IL-12, and increased the expression of IL-10, IL-4, IL-6 and IL-1B. Conversely, treatment with lower concentrations of NA, which bind high affinity alpha adrenoreceptors, increased the expression of IL-4 and TNF-α, and reduced the expression of IL-10, IL-6, IL-5, IL-2 and IL-1B. Decreased sympathetic activation through pharmacological blockade and denervation prevents infiltration of T CD8+ cells and is associated with the autoimmune assault observed in T1DM, possibly through macrophage signalling. The loss of sympathetic innervation observed in T1DM is associated with altered macrophage signalling, increased T CD8+ infiltration and autoimmune assault, but the precise etiology is unclear. Solid lines represent known interactions and dashed lines represent putative interactions.

Albeit only a few studies have investigated the interplay between sympathetic signalling and immune modulation in the pancreas, recent work has supported a role for pancreatic sympathetic innervation in immune modulation and protection from autoimmune diabetes in mice (Figure 6). In one study, pancreatic nerve transection and systemic chemical denervation with 6-OHDA protected against development of diabetes in the rat insulin promoter–lymphocytic choriomeningitis virus–glycoprotein (RIP-LCMV-GP) mouse model of type I diabetes. Simultaneously, islet macrophages were closely associated with sympathetic fibres, expressed adrenergic receptors at high levels and shifted their cytokine production with adrenergic stimulation - in particular, when cultured with NA at high or low concentrations, macrophages were respectively polarized towards M1 (pro-inflammatory) or M2 (anti-inflammatory) phenotypes, and this was attributed to the preferential activation of beta receptors at high NA concentrations (Christoffersson et al., 2020). In contrast, targeted electrical stimulation of sympathetic innervation to pancreatic lymph nodes but not islets inhibited inflammatory cytokine production and progression of diabetes in non-obese diabetic (NOD) mice (Guyot et al., 2019). These findings have been reproduced to certain extent in humans by a study that quantified sympathetic nerve fibre area from autopsy samples from T1DM and T2DM patients and from control subjects and found that patients with T1DM had severe loss of sympathetic innervation at the islets (Mundinger et al., 2016). In line with these findings, a recent study observed altered adrenergic signalling and loss of sympathetic innervation in individuals with non-diabetic islet autoantibody-positive and T1DM individuals (Campbell-Thompson et al., 2021). Out of the specific diabetes context, one study has also found that sympathetic denervation leads to anti-inflammatory signalling in a dog model of acute necrotizing pancreatitis - in this model, denervation resulted in increased IL-10 and reduced TNF-α and C-reactive protein in the serum, which points towards the role of sympathetic signalling in regulating more general inflammatory responses in the pancreas (Sun et al., 2015). Other study has suggested that this regulating role is distributed across several abdominal organs innervated by sympathetic nerves, such as pancreas, liver, spleen, stomach and intestine (Martelli et al., 2019). While further work is needed to dissect the exact contributions of sympathetic innervation in the pancreas, lymph nodes and potentially other abdominal organs to immune function and the regulation of pancreatic endocrine function pancreatic, current evidence support the close interactions between innervation, metabolism and immune function in the pancreas, similarly to what has been observed in other metabolically important organs.

### Neuroimmunometabolism in Liver

**Figure 7:**
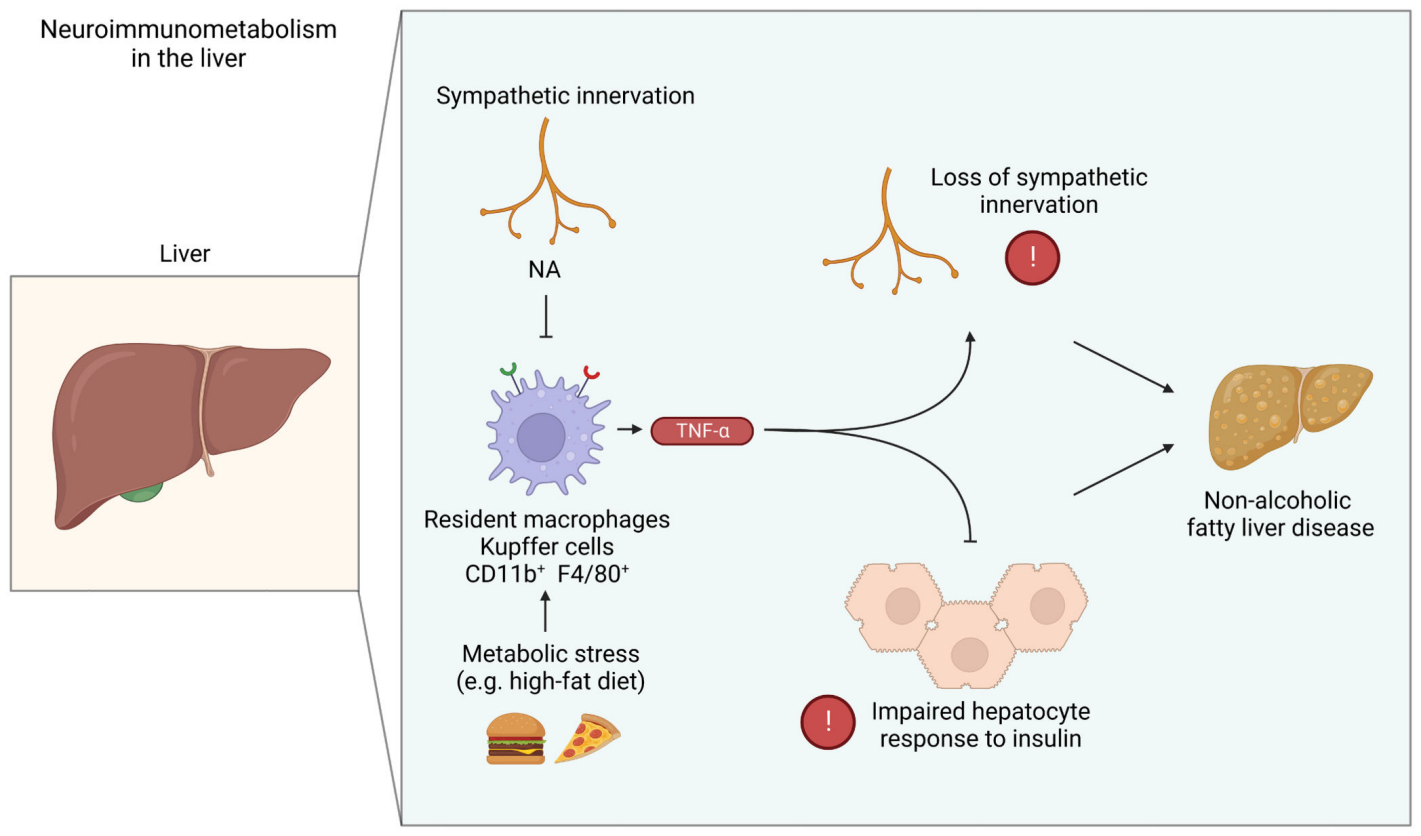
Neuroimmunometabolism in the liver. Noradrenaline (NA) is released by sympathetic nerve terminals within the liver. Under metabolic stress (e.g. consumption of a high-fat diet), impaired sympathetic activity is associated with increased production of TNF-α by macrophages/Kupffer cells, which is followed by impaired hepatocyte response to insulin and ultimately sympathetic neuropathy. These responses have been widely associated with the development of non-alcoholic fatty liver disease (NAFLD). Administration of anti-TNF-α neutralizing antibodies was sufficient to completely reverse the loss of sympathetic axons, highlighting the interplay between sympathetic signalling and inflammatory signalling in metabolic liver disease.

In addition to hepatocytes, the liver contains many immune cells including resident B lymphocytes and Kupffer cells (F4/80+) specialized in phagocytosis and participating in the innate responses. Interestingly, these cells were shown to be innervated by sympathetic fibres and to express adrenergic receptors (Forssmann and Ito, 1977, Tabula Muris et al., 2018). Evidence suggests an important neuro-immune component participating to the development of liver diseases such as non-alcoholic fatty liver disease (NAFLD) (Figure 7). NAFLD is a major comorbidity of obesity that comprises a spectrum of conditions that vary in severity from simple hepatic steatosis to the more aggressive inflammatory form of steatohepatitis (NASH), and which often progresses to liver fibrosis, cirrhosis, and end-stage liver failure (Estes et al., 2018). It is estimated that almost a quarter of the global population presents some form of NAFLD (Younossi et al., 2016), which is due to its close association with obesity and T2DM. Disease progression is caused by chronic low-grade inflammation such as observed in these metabolic disorders (Luci et al., 2020). Autonomic signalling has been long implicated in chronic liver disease. First studies found that surgical hepatic denervation or systemic administration of prazosin, an α-adrenergic antagonist, reduced proliferation of hepatic stem cells in rats after partial hepatectomy (Cruise et al., 1987). Similarly, exaggeration of liver damage by electrical stimulation of hepatic sympathetic nerves and administration of NA or adrenaline was also observed and inhibited by administration of alpha-agonists (Iwai and Shimazu, 1996). This was accompanied by the observation that human patients with cirrhosis presented with autonomic dysfunction, which is related to disease severity (Dillon et al., 1994). The specific role of adrenergic receptors was clarified by a series of studies that showed that pharmacological activation of β- and inhibition of α- receptors had anti-apoptotic effects and reduced overall liver injury in mouse models (André et al., 1999, Oben et al., 2003, Dubuisson et al., 2002). More recent studies systematically reproduced these findings directly and indirectly in the context of obesity and NAFLD - for instance, one study showed that administration of beta-antagonist propanolol was related to worsened liver injury in a mouse model of NAFLD (McKee et al., 2013). It has been also found that diet-induced hepatic steatosis is associated with significant sympathetic overactivity in the liver (Hurr et al., 2019), and that pharmacological blockade with 6-OHDA or phenol-based surgical denervation of the portal sympathetic bundle reduces such abnormal lipid accumulation by diminishing liver free-fatty-acids uptake (Hurr et al., 2019). These findings have been translated to human patients, in which indirect clinical measures of increased sympathetic function have been strongly associated with NAFLD in multiple studies (Clough et al., 2020, Targher et al., 2021, Jung et al., 2021). More recently, Adori *et al*. used three-dimensional immunoimaging to evaluate the liver sympathetic innervation in samples from NAFLD patients, and revealed increased sympathetic axonal sprouting associated with mild nerve degeneration in the early stages of disease and a significant loss of sympathetic innervation in advanced steatohepatitis (Adori et al., 2021). They also showed that chronic sympathetic overactivity is a key factor in the phatogenesis of NAFLD. Another recent study by Liu *et al.* (Liu et al., 2021) further elucidated the interaction between the SNS and immune function in the liver. They found that mice with diet-induced obesity presented profound loss of sympathetic innervation after 20 weeks of high-fat diet (HFD) - interestingly, this effect was partly mitigated by a ketogenic HFD. Additionally, diet-induced liver neuropathy was shown to be completely reversible after 4 weeks of caloric restriction. It was also reported that these effects were caused by TNF-α secreted by CD11b^+^ F4/80^+^ macrophages/Kupffer cells, and that administration of anti-TNF-α neutralizing antibodies was sufficient to reverse loss of sympathetic axons. Analogous to observations in adipose tissue (Pirzgalska et al., 2017), most of these immune cells were then found to be adjacent to sympathetic axons in the liver. However, the outcome of differential signalling of α- or β-adrenergic receptors in the immune response to metabolic stress remains unknown, representing a promising direction of study. Together, these data suggest that a bi-directional relationship exists between nerves and resident immune cells of the liver, with the nervous system affecting their functions and the immune cells affecting nerve integrity. Additional studies are needed to better define how precise neuro-immune interactions contribute to the pathogenesis of metabolic liver disorders and whether they represent potential pharmacological targets.

### Neuroimmunometabolism in Adipose Tissues

**Figure 8:**
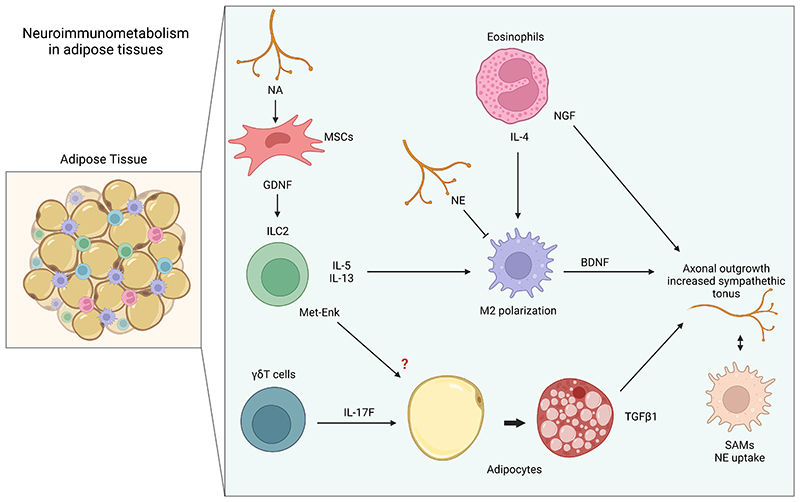
Neuroimmunometabolism in adipose tissue. Noradrenaline (NA) is released by sympathetic nerve terminals within adipose depots. Adipose mesenchymal stromal cells (MSCs) neighbouring these terminals are activated via the *β2* adrenergic receptor to produce glial-derived neurotrophic factor (GDNF), which controls the activity of ILC2s in visceral fat. In turn, ILC2s produce type 2 innate cytokines and Met-enkephalin (Met-Enk), whose direct role in browning is debated. Type 2 innate cytokines act on macrophages to induce M2 polarization, which is also induced by eosinophil-derived IL-4 and inhibited by direct NA signalling to macrophages. M2 polarization is associated with BDNF secretion by macrophages, which leads to axonal outgrowth and increased sympathetic tone. Eosinophils producing nerve growth factor (NGF) can also stimulate sympathetic axonal outgrowth. T cells stimulate *beiging* via IL-17F signalling to adipocytes, which produce transforming growth-factor beta-1 (TGF) to stimulate axonal outgrowth. Sympathetic neuron-associated macrophages (SAMs) contribute to obesity and negatively control sympathetic tone by importing and metabolizing NA.

Immune cells reside in adipose tissues and perform an important homeostatic role. In obesity, however, a chronic low-grade inflammatory environment is established in the metabolic tissues. Adipose tissue macrophages (ATMs) increase from 10% of the adipose tissue resident immune cells to 50% and tend to change from M2 anti-inflammatory to a M1 pro-inflammatory phenotype, producing proinflammatory cytokines including IL-6, IL-1β and TNF-alpha and paracrine factors. Proinflammatory ATMs were indirectly associated with insulin resistance, vascular remodelling, and increased adiposity, but over the last decade their role as architects of lipolytic signalling has been increasingly accepted. Indeed, among the tissues discussed in the present review, the adipose tissue is the one in which immune populations and their interplay with autonomic signaling are most extensively described (Figure 8).

Initial studies suggested that ATMs directly synthesised NA to stimulate thermogenesis, but multiple studies failed to find any intracellular apparatus to synthesise NA in vivo and in vitro. In 2017, Pirzgalska et al reported a new subclass of macrophages that reside in direct contact with sympathetic neurons and adipocytes in humans and mice – sympathetic associated macrophages (SAMs) (Pirzgalska et al., 2017). Crucially, SAMs expressed noradrenaline transporter (Slc6a2) and monoamine oxidase-A enzyme, but no NA synthesising enzymes directly. Optogenetic activation of SNS increased SAM’s uptake of NA, and tissue specific deletion of Slc6a2 prevented this, while also increasing BAT content, promoting *beiging* of WAT and causing a significant and sustained weight loss in obese mouse models (Pirzgalska et al., 2017). Likewise, Wolf et al. (Wolf et al., 2017) reported a specialised population of macrophages in BAT that control tissue innervation. Explicitly, they proved that the deletion nuclear transcription regulator methyl-CpG binding protein 2 (Mecp2) in specific tissue macrophages provokes a reduction in sympathetic innervation in BAT and this led to obesity by altering body composition because of alterations thermogenesis. Other myeloid cells appear to promote SNS activity. In 2020, Blaszkiewicz and colleagues reported a set of myeloid cells residing in subcutaneous WAT that expressed adrenergic receptors and secrete BDNF. Silencing of these macrophages caused a ‘genetic denervation’ of iWAT, resulting in increased adiposity, decreased energy expenditure and blunted UCP1 response to cold stimulation, suggesting these macrophages are vital in regulating adipose innervation and sympathetic remodelling, as well as maintaining homeostatic control of metabolism (Blaszkiewicz et al., 2019).

Conversely, SNS activity also influences the activity of local immune cells. Cardoso *et al.* (Cardoso et al., 2021) report that chemogenetic activation or ablation of gonadal WAT SNS, respectively, increases or decreases local adipose group 2 innate lymphoid cells (ILC2) function via a β2 dependent mechanism to stimulate the production of glial-derived neurotrophic factor (GDNF) by mesenchymal stromal cells (MSCs) (Cardoso et al., 2021). ILC2s were also shown to be present in human WAT and to produce methionine-enkephalin (Met-Enk) (Brestoff et al., 2015). ILC2s production of Met-Enk and of type-2 cytocines was shown to be reduced in mice treated with 6-OHDA (Cardoso et al., 2021). A study by Brestoff *et al.* showed that Met-Enk in vivo administration promoted *beiging* of WAT, however, an indirect effect is possible as opioids are potent respiratory supressors that also emaciate. To test for a direct effect *in vitro*, they differentiated the stromal vascular fraction (SVF) onto adipocytes and incubated with Met-Enk to onserve an upregulation of *Ucp1* (Brestoff et al., 2015). However, adipocyte cultures differentiated from SVF ca contain additional cell types that also express opioid receptors *Ogfr* and *Oprd1*, which the authors indeed detected in whole BAT and iWAT by rtPCR - with *Ogfr* being shown to be preferentially expressed in BAT and *Oprd1* in WAT. In line with this possibility, publicly available single-nuclei RNA sequencing (snRNAseq) data from WAT (Rajbhandari et al., 2019) shows that *Oprd1* and *Ogfr* is expressed in multiple immune populations from the SVF, while adipocytes express only *Ogfr*. In snRNAseq data from BAT adipocytes (Song et al., 2020), *Ogfr* was very lowly expressed and *Oprd1* was not expressed. *Oprm1,* another opioid receptor, was not expressed in adipocytes from either BAT or WAT. Thus, Met-Enk actions could be indirect and mediated by immune cells instead of acting directly on adipocytes. Additionally, it is still unknown which of the opioid receptors are effectively targeted by Met-Enk to result in the observed *beiging* effects, and whether this is mediated by additional populations. Intracellularly, it is also unclear how opioid signalling in adipocytes would lead to *beiging*.

Other immune cell types have also been implicated in the neuroimmune control of adipose tissue. Eosinophils, for instance, have been shown to promote macrophage polarization to an M2 state by production of IL-4 and to coordinate the plasticity of axonal outgrowth by producing nerve growth factor (NGF) (Meng et al., 2022). Paralelly, γδT-cells have also been shown to contribute to *beiging* and axonal outgrowth by IL-17 signalling to adipocytes (Hu et al., 2020). Ultimately, adipocytes produce transforming growth-factor beta-1 (TGFβl) during *beiging* and browning, stimulating axonal outgrowth (Hu et al., 2020, Krieglstein et al., 2002). Thus, by multiple pathways, immune cells play a key role in the regulation of SNS activity in the adipose tissue, either by direct stimulation of axonal outgrowth and sympathetic tonus by the liberation of growth factors, or by the upregulation of BDNF-producing M2 macrophages and inhibition of the M1 proinflammatory phenotype. Therefore, strategies that modulate the balance between these macrophage states are attractive therapeutic interventions to be explored.

Collectively, these findings establish a novel homeostatic role of tissue macrophages and other immune cells that can control sympathetic innervation. Any disruption between this neuroimmune crosstalk might impact the nerve integrity and tissue function with a considerable final effect on metabolism.

## Anti-Obesity Therapies: Sympathomimetics vs Sympathofacilitators

Obesity is a leading cause of morbidity and mortality globally, but until recently, anti-obesity pharmaceuticals have had poor therapeutic efficacy and tolerance. The prolipolytic signalling of the sympathetic nervous system has attempted to be exploited by sympathomimetics - compounds that stimulate sympathetic nerves by inhibition of MA uptake or direct stimulation of peripheral adrenoceptors (Westfall, 2009). They have potent anti-obesity effects but are plagued by cardiovascular toxicity and propensity for addiction, limiting their clinical tolerability (Finer, 2002, Araujo and Martel, 2012).

Classically, the mechanism of action of sympathomimetic-induced weight loss was thought to be central, by suppressing appetite and promoting locomotion. The addictive properties of sympathomimetic are due to nigrostriatal monoamine release; cardiovascular toxicity is thought to be caused by a combination of central and peripheral stimulation. This view was challenged by Mahú and colleagues, who brought attention to the contribution of the brain on the peripheral readouts of sympathomimetics (Liu and Varner, 1996, Hassan et al., 2015), while proposing that lipolytic action can emerge from peripheral mechanisms (Mahu et al., 2020). Amphetamine (AMPH) administration in vitro facilitates sympathetic firing as measured via intracellular calcium imaging and genetic ablation of sympathetic neurons blocked the protective effect of AMPH treatment against diet induced obesity. Administration of PEGyAMPH, a modified form of amphetamine that is too large to cross the BBB, increased lipolysis, thermogenesis, and protected mice against obesity when given a HFD challenge, without inducing hypophagia or hyperkinesis. Moreover, this effect appears to be dependent on heat dissipation and ß2 adrenergic signalling – elevated ambient temperature and ß2 antagonist butoxamine inhibited the sympathofacilitator and weight-loss effect of PEGyAMPH. Crucially, PEGyAMPH treatment showed no effects on cardiovascular function, unless they were introduced intracerebroventricularly (Mahu et al., 2020). Although molecular target(s) of PEGyAMPH are unknown, a new class of anti-obesity drugs that facilitate the activity of sympathetic neurons is proposed – ‘Sympathofacilitators’. This class is distinct from the sympathomimetic class in that the effect lies pre-synaptically and is contingent on an incoming cholinergic input originating from spinal pre-ganglionic neurons, which in turn receive descending inputs from higher order circuitry that commands on metabolic control. Sympathofacilitators may amplify the weakened descending neuronal signals originating from a blunted action of leptin in the brain which characterizes a state of leptin resistance (Figure 5). This state emerges when the neuroendocrine loop of leptin escapes homeostasis, and the ratio of afferent and efferent signals are out of proportion. Hyperleptinemia is required for the development of leptin resistance (Knight et al., 2010) and reducing leptin levels with neutralizing antibodies or temporally controlled genetic loss of function has been proven to restore homeostasis (Zhao et al., 2019). Normalizing excessive afferent leptin can be seen as the reciprocal approach to boosting the weaked efferent arm of the neuroendocrine loop via a Sympathofacilitators drug (Figure 5). Unlike the heart, metabolic organs such as WAT/BAT and liver are not counter opposed by parasympathetic innervation, which may in turn account for PEGyAMPH’s cardioprotective effect.

ß3-adrenoceptor agonists have also received attention due to their ability to promote BeAT differentiation and formation in rodents, but poor selectivity and systemic administration of early ß3 agonists caused intolerable side-effects (Arch, 2011).

Recent attention emerged surrounding Mirabergon, a specific ß3 agonist already used to treat urinary urge incontinence. In 2015, Cypess and colleagues measured 18F-FDG uptake in PET scans to show a 200mg dosing regimen of Mirabegron increases human BAT metabolic activity, while also increasing BAT glucose uptake and increasing resting metabolic rate by 230±40 kcal/day (Cypess et al., 2015). Subsequent trials indicate improved glucose homeostasis in obese subjects (Finlin et al., 2020), HDL cholesterol and insulin sensitivity (O’Mara et al., 2020). These studies all in younger populations with average ages from 27.5 (O’Mara et al., 2020) to 54.8 (Finlin et al., 2020). As human BAT activity is inversely correlated with age (Cypess et al., 2009) and in mouse models, ß3 adrenoceptor expression is reduced in diabetic and obese subjects (Collins et al., 1994), there is a risk that those with the most advanced metabolic syndromes may not be able to respond to Mirabegron. Synergistic therapy regimes of Pioglitazone, a PPARy agonist that upregulates ß3-adrenoceptor expression and agonist responsiveness in preclinical models, do not have evidence of improved metabolic outcomes compared to Mirabegron alone (Kern et al., 2020, Finlin et al., 2021). The role of human β3-adrenoceptor has recently been questioned by studies showing a lack of effect of Mirabegron on human thermogenesis, which the study demonstrated to be driven by β2 adrenergic signalling (Blondin et al., 2020).

## Conclusions & future perspectives

Relative to brain research, sympathetic neuroscience is lagging several decades behind - owing to the dispersed and difficult anatomical access and technical limitations that the developments we envision will likely surpass. These will be necessary to overcome the conceptual barrier that stagnated in the era of adrenergic receptor pharmacology, which solely accounts for post-synaptic adrenergic targeting in organs. Although such models account for the action of a few adrenergic drugs that are routinely used in the clinic (such as mydriatics, bronchodilators or vasopressors), sympathetic nerves are seldom considered as a neural circuit, whereby pre-synaptic manipulations of a node within the network might not be predicted by post-synaptic adrenoceptor agonism or centrally acting sympathomimetics which have a widespread top-down action. Moreover, the assessment of adrenoceptor subtype expression across cell types mainly originates from bulk tissue techniques that do not account for expression by resident immune cells. Only single nuclei sequencing has parsed apart tissue heterogeneity, particularly that of WAT&BAT, which cannot be analyzed by conventional cell sorting.

Moreover, the linearity of post-synaptic agonism falls apart as adrenoceptors are also expressed in sympathetic neurons, suggesting that feedforward or feedback circuits may exist among ganglia. Whether intra- and inter ganglionic circuitry exists is an open question, despite the highly suggestive TH+ axon bundles between every two ganglia along the paravertebral sympathetic chain. Such ganglionic circuits can be influenced by resident immune cells that cleanse norepinephrine or produce neuromodulators such as opioids. Thus, the field needs to rise from the era of post-synaptic adrenoceptor pharmacology to that of neural networks. As such, the classic view that SNS is responsible for the ‘fight or flight’ response antagonised by the PNS is an incomplete description of autonomic metabolic control in light of the modern neuronal network theory. Not only do adipose tissues and liver (although still under discussion) receive innervation exclusively from the SNS, but PNS in the heart can act as a cardioprotective buffer whilst sympathofacilitator drugs exert effective control of metabolic tissues to facilitate weight loss and restore the homeostasis of the neuroendocrine loop of leptin action.

Current molecular genetic technologies widely used in the brain have had limited success with SNS circuits. Dissections of the murine paravertebral sympathetic chain of ganglia are complicated, and retrograde tracing with a non-replicative non-polysynaptic virus has low transduction efficiency, perhaps due to the cleansing of viral particles by immune cells resident in sympathetic tissues. PRV retrograde tracing pioneered mapping the efferent postganglionic sympathetic innervation of BAT, which seems consensual across species. However, the innervation of WAT is more variable across species and the location of the fat depot. Similarly, the origin of the sympathetic innervation of the liver is inconsistent and seems restricted to the perivascular space. Unlike liver and adipose tissues, and more aligned with conventional autonomic paradigms, the pancreas possesses both sympathetic innervation and parasympathetic innervation with opposing effects. Differential sympathetic activation would be advantageous for selective targeting and treatment of organ-specific dysfunction, including immune and metabolic pathologies. Except for central leptin action, it is debatable whether manipulations of upstream brain circuits would recruit a top-down response that is cardioneutral and specific to metabolic organs. Centrally acting sympathomimetics such as amphetamines and MCR8 agonists recruit widespread and cardiotoxic sympathetic drive, indicating that higher-order manipulations may not be more targetted than those of lower order at the postganglionic level. Drawing a parallel with the organization of other descending axes such as sensory-motor circuits: lesions or stimulations of higher order brain structures produce effects that are more widespread relative to peripheral manipulations, for instance, when comparing the sensory-motor deficits originating from the stroke of the central medial artery to those arising from peripheral lesions. This organizational principle could also apply to the sympathetic axis, whereby peripheral postganglionic targeting may be more specific than higher-order pre- sympathetic central circuits. Thus, manipulations of sympathetic postganglionic neurons may be a means to achieve metabolic control while identifying neural circuits, or nodes within them, that preserve cardiovascular health. Critical to this endeavour will be to generate transgenic CRE/DRE driver lines for subpopulations of neurons that could be systematically used as molecular handles for remote activation/inhibition via minimally invasive technologies, such as chemogenetics or magnetogenetics, for a causal assessment of the exact neuronal pathways of the sympathetic-metabolic axis. Realistic maps with single-neuron resolution akin to the MouseLight Project are still missing. A functional understanding of SNS circuitry in-vivo will require the development of non-invasive genetically encoded activity reporters that can be sensed in deep-seated sympathetic ganglia – possibly capitalizing on optoacoustic as an enabling technology.

Obesity-induced sympathetic neuropathy is a cause and effect of metabolic disease – weakening the sympathetic efferent arm in the neuroendocrine loop of leptin action. Reversing obesity-induced sympathetic neuropathy may be a step towards re-establishing body weight homeostasis, and this will require deciphering the cross-talk among sympathetic-resident immune cells. This prospect promises “sympathoprotective” immunotherapeutic targets and could offer a new generation of safe and effective anti-obesity treatments.

## BOX1 Adrenergic receptor types in pancreas, liver and adipose tissues.

The adrenergic receptors (AR) are a class of G protein-coupled receptors expressed in a wide number of cells. These receptors are classified as α1, α2, and β, each of which is subdivided into three subtypes. Regard et al. (Regard et al., 2008) analysed the pattern of adrenergic/muscarinic receptors expression in different organs. In pancreas, α2AR in pancreatic ß-cells inhibit insulin secretion and increases glucagon secretion (Fagerholm et al., 2011). Additional adrenergic receptors (α2B and α2CAR) (Amisten et al., 2013, Thor, 2022) have also been reported on murine pancreatic ß-cells and delta cells. Both ß1 and ß2 receptors have been reported on pancreatic alpha and beta cells, and ß2 agonists increase insulin release in mice (Panagiotidis et al., 1993) and dogs, and stimulate glucagon release in dogs (Kaneto et al., 1975). In liver, the main adrenergic receptors are α1 and ß2ARs and catecholamine- stimulated glycogenesis seems to occur primarily via ß2AR (Rizza et al., 1980, Kulcsar-Gergely and Kulcsar, 1989, Katz et al., 1993). Also, a population of ß1AR have been reported in rat liver (Fraeyman et al., 1992, Cardani and Zavanella, 2001). In murine white and brown adipose tissues, the three βAR have been identified but the ß3AR is the predominant in both (Collins et al., 1994). NA released from the SNS terminals in the adipocytes activates ß3AR and stimulates lipolysis. Specifically, in BAT this stimulates the uncoupling protein 1 (UCP1) activity and increases energy expenditure. In WAT, the ß3AR activation can lead to the browning of white adipocytes too, contributing to thermogenesis. In addition, the β1AR expressed in pre-adipocytes can be stimulated by NA and push UCP1 expression and beige adipogenesis (Jiang et al., 2017). α1 and α2AR have been detected in white and brown adipocytes but their functionality is still unclear (reviewed in Michel *et al* (Michel et al., 2019)). In humans, β2AR is expressed in BAT and mediates thermogenesis (Blondin et al., 2020).
